# 2025 US Public Health Service Guidelines for the Management of Occupational Exposures to Human Immunodeficiency Virus and Recommendations for Post-exposure Prophylaxis in Healthcare Settings

**DOI:** 10.1017/ice.2025.10254

**Published:** 2025-09

**Authors:** Aaron D. Kofman, Kimberly A. Struble, Walid Heneine, Britt Gayle, Marie A. de Perio, Devon L. Okasako-Schmucker, Christine N. So, Laura E. Anderson, Erin C. Stone, David K. Henderson, David T. Kuhar

**Affiliations:** 1 Division of Healthcare Quality Promotion, National Center for Emerging and Zoonotic Infectious Disease, Centers for Disease Control and Prevention, Atlanta, GA, USA; 2 Division of Antiviral Products, Center for Drug Evaluation and Research, Food and Drug Administration, Silver Spring, MD, USA; 3 Division of HIV Prevention, National Center for HIV, Viral Hepatitis, STD, and TB Prevention, Centers for Disease Control and Prevention, Atlanta, GA, USA; 4 HIV/AIDS Bureau, The Health Resources and Services Administration, Rockville, MD, USA; 5 Office of the Director, National Institute for Occupational Safety and Health, Centers for Disease Control and Prevention, Cincinnati, OH, USA; 6 Chenega Enterprise Systems and Solutions, Chesapeake, VA, USA; 7 Hospital Epidemiology Service Clinical Center, National Institutes of Health, Bethesda, MD, USA

## Abstract

These guidelines update the 2013 “Updated US Public Health Service (PHS) Guidelines for the Management of Occupational Exposures to Human Immunodeficiency Virus and Recommendations for Postexposure Prophylaxis,” hereafter referred to as the 2013 PHS Guidelines.^1,2^ The availability of new medication options, new information on the window of detection for different human immunodeficiency virus (HIV) tests, and the risk of transmission from people with undetectable viral loads prompted this update. The primary intended audience for these recommendations remains anyone involved in the provision of HIV post-exposure management to healthcare personnel (HCP).

The U.S. Centers for Disease Control and Prevention assembled a working group of representatives from federal agencies in the U.S. Department of Health and Human Services (HHS) who identified the priority topics for update and conducted systematic literature reviews to formulate recommendations (see Appendix). All recommendations were reviewed by the Healthcare Infection Control Practices Advisory Committee (HICPAC) at public meetings, and by a non-consensus forming panel of external experts. New evidence-based recommendations are developed using the Grading of Recommendations Assessment, Development, and Evaluation (GRADE) framework and classified according to the HICPAC recommendation scheme when evidence supported recommendation development.^3,4^ Other recommendations in this document are classified as good practice statements according to the criteria set forth by GRADE.^5^ The working group solicited additional feedback on recommendations from relevant agencies, subject-matter experts, and the public.

Recommendations that have changed since the 2013 PHS guidelines include:

- new antiretroviral drug regimens for post-exposure prophylaxis (PEP);
- a shortened duration of post-exposure follow-up HIV testing;
- elimination of routine laboratory tests for antiretroviral drug toxicity; and
- considerations for PEP for HCP with exposures to source patients with undetectable viral loads.

Important strategies in the principles of exposure management remain: primary prevention strategies; the prompt reporting and management of occupational exposures; adherence to recommended HIV PEP regimens when indicated; the role of expert consultation in management of exposures; and follow-up of exposed HCP.

## Introduction

Preventing exposures to blood and body fluids (i.e., primary prevention) is the most important strategy for preventing occupationally acquired human immunodeficiency virus (HIV) infection in healthcare settings and is grounded in adherence to the principles of Standard Precautions^6^ by healthcare personnel (HCP) and their institutions. For instances in which an occupational HIV exposure may have occurred, appropriate management with post-exposure prophylaxis (PEP) is an additional element of workplace safety, and its provision is required by the U.S. Occupational Safety and Health Administration (OSHA).^7^ These guidelines provide updated recommendations on the management of occupational exposures to source patients with known or suspected HIV infection in healthcare settings. These recommendations are aimed at any persons involved in the provision of HIV post-exposure management to HCP, including but not limited to the leaders and staff of occupational health services (i.e., employee health services), emergency and urgent care providers, and primary care providers. HCP, infection prevention, and control staff, and individuals in human resources departments will also benefit from these recommendations.

Key updates in these guidelines include: (1) preferred and alternative antiretroviral therapy (ART) for use as PEP regimens, (2) recommendations for HCP exposures to patients with undetectable viral loads, (3) recommendations for HCP who are on pre-exposure prophylaxis (PrEP) at the time of exposure, and (4) timing for conclusion of HIV follow-up testing after exposure and other laboratory diagnostic test considerations. Since the 2013 Public Health Service (PHS) Guidelines^1^, the U.S. Food and Drug Administration (FDA) approved several new antiretroviral agents for the treatment of HIV, including second-generation integrase inhibitors with higher barriers to resistance, comparatively fewer side effects, and more convenient dosing schedules than older agents. In addition, new data were published on the effectiveness of HIV tests in detecting early infection and on the risk of transmission from source patients with undetectable viral loads. In light of these new developments, a U.S. PHS working group was convened by CDC to review new data on HCP with occupational exposures to HIV and update recommendations where the evidence has changed since 2013.

As in the 2013 PHS Guidelines, this update continues to emphasize: (1) primary prevention of occupational exposures; (2) prompt reporting and management of occupational exposures and, if indicated, initiation of PEP as soon as possible after exposure; (3) selection of PEP regimens that have the fewest side effects and that are best tolerated by recipients; (4) attention to potential interactions involving both drugs included in PEP regimens and other medications that PEP recipients might be taking; (5) consultation with experts on PEP management strategies for complex cases such as assistance with determining whether an exposure has actually occurred, or selecting PEP regimens for cases where the source patient is known or suspected to have extensive HIV mutations associated with antiretroviral resistance; (6) HIV testing of source patients (without delaying PEP initiation in the exposed provider) using methods that produce rapid results; and (7) counseling and follow-up of exposed HCP.

Recommendations on the management of occupational exposures to hepatitis B virus and/or hepatitis C virus (HCV) have been published previously^8,9^ and are not included in these guidelines. Recommendations for nonoccupational (e.g., sexual, pediatric, and perinatal) HIV exposure also have been published previously and are updated separately.^10–12^

## Methods

In February 2022, the Centers for Disease Control and Prevention (CDC) convened an interagency U.S. PHS working group to plan and prepare an update to the 2013 PHS guidelines.^1^ The PHS working group comprised representatives from the CDC, the FDA, the Health Resources and Services Administration (HRSA), and the National Institutes of Health (NIH). Names, credentials, and affiliations of the PHS working group members are listed as the byline of these guidelines. The working group reviewed the 2013 PHS guidelines to identify gaps in recommendations, those recommendations that required update or change, and those that did not require update and should remain as standard of care. Based upon this review, CDC experts and methodologists developed five key questions to guide the systematic literature review as follows:

1. What is the balance of benefits and risks of prescribing dolutegravir and/or bictegravir-containing regimens compared to raltegravir plus emtricitabine/tenofovir for use as first-line PEP among HCP with an occupational exposure to HIV?
2. What is the balance of benefits and risks of prescribing intramuscular (IM)-administered cabotegravir-based ART compared to the standard of care of use as an alternative PEP regimen among HCP with an occupational exposure to HIV?
3. What is the interval following an occupational HIV exposure after which no benefit is gained by administering PEP to HCP?
4. What is the risk of a patient with an undetectable HIV viral load transmitting to HCP following an occupational percutaneous and/or mucous membrane exposure?
5. What is the window of time until HIV antibodies can be detected using a fourth-generation HIV antigen/antibody (Ag/Ab) test, among HCP with an occupational exposure?

CDC methodologists (E.C.S., D.O.S., and C.N.S.) conducted a systematic literature review according to PRISMA reporting guidelines, the body of evidence was evaluated using the GRADE approach (see Appendix),^13^ and new recommendations were developed using HICPAC methods.^3^ A working group meeting was held in February 2023 to discuss the systematic literature review results and develop draft recommendations. Recommendation categories and their definitions with bolded rationale, are found in Table 1. Evidence-based recommendations were categorized as Recommendations or Conditional Recommendations,^3^ and recommendations that were not informed by systematic literature reviews were categorized as Good Practice Statements^5^ according to the rationales found in Table 1. All draft recommendations were shared with the nonoccupational HIV PEP working group to harmonize recommendations between guidelines where applicable. The complete methods including rationale for recommendations, collected expert evidence, evidence to decision-making frameworks, and the results of the systematic literature review are available in the Appendix.

**Table 1.** Recommendation categorization framework

Category	Criteria/ Rationale
**All Statements**	The statement is: • clear and actionable, and • the message is necessary with regard to actual healthcare practice.
**Statements developed using systematic review evidence**	
**Recommendation**	A new statement supported by **High or moderate confidence** in systematic review evidence indicating benefit or harm
**Conditional Recommendation**	A new statement supported by **Low confidence** in systematic review evidence indicating benefit or harm
	A new statement supported by **High or moderate confidence** in systematic review evidence suggesting benefit or harm
**Statements developed using alternate sources of evidence**	
**Good Practice Statement** ^5^	Good Practice Statements (GPS) are developed when: • Implementing the GPS results in a large net-positive consequences. • Collecting and summarizing the evidence is a poor use of a guideline panel’s limited time, energy or resources.
	The rationale for a GPS may be that it is • An **Existing CDC Recommendation** that does not require a change in meaning or update at this time, or a new statement supported by o high or moderate confidence in a well-documented rationale connecting **Indirect Evidence** from animal, pharmacokinetic mechanism of action, or basic science studies, or o systematically collected **Expert Experience** ^5^ for instances when evidence collection would be a poor use of the panel’s time and resources.

The PHS working group presented to a non-consensus forming panel of external subject-matter experts in July 2023. The expert panel consisted of professionals in academic medicine considered to be experts in the treatment of people with HIV, the use of antiretrovirals for PEP, occupational medicine, hospital epidemiology, and HIV laboratory diagnostic tests. Names, credentials, and affiliations of the expert panel of consultants are listed in “Expert Panel Consultants” section. Feedback from the expert panel was incorporated, and the updated draft recommendations were presented to HICPAC in August 2023 during a public meeting, at which there were no suggested edits or recommendations from either the committee or the public.^14^ In October 2024, additional peer review was solicited from experts in the field of HIV and infection prevention, in compliance with requirements of the U.S. Office of Management and Budget (OMB) for influential scientific assessments. Names, credentials, and affiliations of the OMB peer review panel are listed in the “OMB Peer Review Panel” section. Feedback was incorporated into the finalized guidelines.

## Summary of recommendations

*Recommendation categorization definitions and supporting rationale are listed in* Table 1 *. All Existing Recommendations are from the 2013 Guidelines. Additional details can be found in Appendix Table S2.*

### Management of HCP with an occupational exposure to HIV

1. Educate HCP to report occupational exposures to blood and body fluids as soon as possible to their occupational health service. **Good Practice Statement**, Existing Recommendation
2. Initiate PEP as soon as possible, up to 72 hours following the occupational exposure to HIV. **Good Practice Statement**, Existing Recommendation
3. When considering initiation of PEP after 72 hours following occupational exposures thought to represent a high risk of transmission, consult a provider with expertise in HIV treatment^*^ (see Box 1). **Good Practice Statement**, Existing Recommendation
4. Prescribe PEP regimens for a duration of 28 days. **Good Practice Statement**, Existing Recommendation
5. Determine the HIV status of the source patient whenever possible to guide appropriate use of PEP. **Good Practice Statement**, Existing Recommendation
6. Do not delay administration of PEP while waiting for information regarding the source patient’s HIV status. **Good Practice Statement**, Existing Recommendation
7. Discontinue PEP and HIV follow-up testing if the source patient is determined to be HIV negative. **Good Practice Statement**, Existing Recommendation
8. Provide counseling to exposed HCP in accordance with CDC recommendations for HCP with occupational exposures^15^, and including the:
  - Importance of adherence to PEP;
  - Use of precautions (i.e., use of barrier contraception, avoidance of blood or tissue donations) to prevent secondary transmission until the final HIV test post-exposure (see **Laboratory Testing of Exposed HCP**);
  - Drugs^16^ that should not be taken with PEP or require dose or administration adjustments, side effects of prescribed PEP, and measures (including pharmacologic interventions) that may assist in minimizing side effects; and
  - Importance of clinical evaluation if any acute symptoms (i.e., side effects of prescribed PEP, symptoms of acute HIV) develop prior to the final HIV test post-exposure (see **Laboratory Testing of Exposed HCP**).
  - Good Practice Statement, Existing Recommendation
9. Re-evaluate exposed HCP within 72 hours after occupational exposure to assess for further counseling needs and PEP tolerability. **Good Practice Statement**, Existing Recommendation
10. Consult a provider with expertise in HIV treatment^*^ for HCP unable to take the initial PEP regimen due to intolerance or toxicity (see Box 1). **Good Practice Statement**, Existing Recommendation
11. Refer HCP determined to have HIV infection to a provider with expertise in HIV treatment^*^. **Good Practice Statement**, Existing Recommendation

Box 1. Resources for expert consultation for Human Immunodeficiency Virus (HIV) Post-exposure Prophylaxis (PEP)

- Expert consultation can be made with local experts or through the following resources:
- Antiretroviral Pregnancy Registry at http://www.apregistry.com; telephone: 800-258-4263; fax: 800-800-1052; email: sm_apr@apregistry.com
- National Clinician Consultation Center (UCSF) Post-Exposure Prophylaxis Hotline at 888-448-4911.
- FDA (for reporting unusual or severe toxicity to antiretroviral agents): http://www.fda.gov/medwatch; telephone: 800-332-1088
- The CDC’s Cases of Public Health Importance (COPHI) coordinator (for reporting HIV infections in HCP and failures of PEP) at telephone number 404-639-2050.
- HIV/AIDS Treatment Information Service at http://aidsinfo.nih.gov/.

### Management of pregnant or breastfeeding HCP with an occupational exposure to HIV

1. Provide post-exposure management to pregnant or breastfeeding HCP following occupational exposure based on the same considerations that apply to any HCP following occupational exposure to HIV (see **HCP with an Occupational Exposure to HIV)**. **Good Practice Statement,** Existing Recommendation
2. Consult a provider with expertise in the treatment of HIV in pregnant or breastfeeding women^*^ (see Box 1). **Good Practice Statement,** Existing Recommendation
3. Counsel pregnant HCP regarding potential risks and benefits of selected PEP agents for the fetus. **Good Practice Statement,** Existing Recommendation
4. Counsel breastfeeding HCP regarding:
  - The high risk of HIV transmission through breast milk should acute HIV infection occur; and
  - The risks and benefits of continuing breastfeeding while taking PEP and being monitored for HIV seroconversion; or interrupting breastfeeding and discarding breast milk until the final HIV test post-exposure (see **Laboratory Testing of Exposed HCP**) to eliminate risk of HIV transmission to the infant.

**Good Practice Statement,** Existing Recommendation

### Selection of agents for HIV PEP

1. Consult Table 2 for initial preferred and alternative PEP regimens for HCP following occupational exposure to HIV.

**Table 2.** Initial Preferred and Alternative Human Immunodeficiency Virus (HIV) Post-exposure Prophylaxis (PEP) Regimens for Healthcare Personnel^§^ ^,^ ^¶^ ^,^ ^**^ ^,^ ^††^

Group	Preferred^§^/ Alternative^¶^	Regimen
**Healthcare personnel without conditions specified below**	**Preferred**	**Integrase Strand Transfer Inhibitors PLUS Two Nucleoside Reverse Transcriptase Inhibitors** • Bictegravir/emtricitabine/tenofovir alafenamide • Dolutegravir PLUS (tenofovir alafenamide OR tenofovir disoproxil fumarate) PLUS (emtricitabine OR lamivudine)
	Alternative	**Boosted Protease Inhibitor PLUS Two Nucleoside Reverse Transcriptase Inhibitors** • Darunavir and cobicistat OR darunavir and ritonavir PLUS (tenofovir alafenamide OR tenofovir disoproxil fumarate) PLUS (emtricitabine OR lamivudine)
**Pregnancy;** **expert consultation recommended** ^¶^	**Preferred**	**Integrase Strand Transfer Inhibitors PLUS Two Nucleoside Reverse Transcriptase Inhibitors** • Bictegravir/emtricitabine/tenofovir alafenamide • Dolutegravir PLUS (tenofovir alafenamide OR tenofovir disoproxil fumarate) PLUS (emtricitabine OR lamivudine)
	Alternative	**Boosted Protease Inhibitor PLUS Two Nucleoside Reverse Transcriptase Inhibitors** • Darunavir and ritonavir (twice daily) PLUS (tenofovir alafenamide OR tenofovir disoproxil fumarate) PLUS (emtricitabine OR lamivudine)
**Moderate renal dysfunction (creatinine clearance 30–49 mL/min); expert consultation recommended** ^¶^	**Preferred**	**Integrase Strand Transfer Inhibitors PLUS Two Nucleoside Reverse Transcriptase Inhibitors** • Bictegravir/emtricitabine/tenofovir alafenamide • Dolutegravir PLUS tenofovir alafenamide PLUS (emtricitabine OR lamivudine^§§^)
	Alternative	**Integrase Strand Transfer Inhibitors PLUS Two Nucleoside Reverse Transcriptase Inhibitors** • Dolutegravir PLUS dose-reduced tenofovir disoproxil fumarate^***^ PLUS (emtricitabine OR lamivudine^§§^) **Boosted Protease Inhibitor PLUS Two Nucleoside Reverse Transcriptase Inhibitors** • Darunavir/cobicistat/tenofovir alafenamide/emtricitabine • Darunavir and ritonavir PLUS (tenofovir alafenamide OR dose-reduced tenofovir disoproxil fumarate^***^) PLUS (emtricitabine OR lamivudine^§§^)
**Severe renal dysfunction (creatinine clearance <30 mL/min) and on hemodialysis;** **expert consultation recommended** ^¶^	**Preferred**	**Integrase Strand Transfer Inhibitors PLUS Two Nucleoside Reverse Transcriptase Inhibitors** • Bictegravir/emtricitabine/tenofovir alafenamide • Dolutegravir PLUS tenofovir alafenamide PLUS (emtricitabine OR dose-reduced^†††^ lamivudine)
	Alternative	**Integrase Strand Transfer Inhibitors PLUS Two Nucleoside Reverse Transcriptase Inhibitors** • Dolutegravir PLUS dose-reduced tenofovir disoproxil fumarate PLUS (emtricitabine OR dose-reduced^†††^ lamivudine) **Boosted Protease Inhibitor PLUS Two Nucleoside Reverse Transcriptase Inhibitors** • Darunavir/cobicistat/tenofovir alafenamide/emtricitabine • Darunavir and ritonavir PLUS (tenofovir alafenamide OR dose-reduced^†††^ tenofovir disoproxil fumarate) PLUS (emtricitabine OR dose-reduced^†††^ lamivudine)
**Severe renal dysfunction (creatinine clearance <30 mL/min; not on hemodialysis)**; **expert consultation recommended** ^¶^		Consult HIV specialist
**Hepatic impairment (Child-Pugh A or B);** **expert consultation recommended** ^¶^	**Preferred**	**Integrase Strand Transfer Inhibitors PLUS Two Nucleoside Reverse Transcriptase Inhibitors** • Bictegravir/emtricitabine/tenofovir alafenamide • Dolutegravir PLUS (tenofovir alafenamide OR tenofovir disoproxil fumarate) PLUS (emtricitabine OR lamivudine)
	Alternative	**Boosted Protease Inhibitor PLUS Two Nucleoside Reverse Transcriptase Inhibitors** • Darunavir and cobicistat OR darunavir and ritonavir PLUS (tenofovir alafenamide OR tenofovir disoproxil fumarate) PLUS (emtricitabine OR lamivudine)
**Hepatic impairment (Child-Pugh C);** **expert consultation recommended** ^¶^		Consult HIV specialist

The regimens below are recommended for initial use as PEP in HCP exposed to HIV. When the recommended preferred regimens are not available, alternative regimens are suitable for use. Although a raltegravir-based regimen is not included in the table due to having a higher pill burden and potential for decreased adherence, it remains an efficacious option for occupational post-exposure prophylaxis if neither preferred nor alternative regimen(s) can be prescribed.

§ Preferred PEP Regimens are categorized as Recommendations supported by Moderate Confidence in the evidence.

¶ Alternative PEP Regimens and consultation recommendations are categorized as Good Practice Statements based on Existing Recommendations.

** The following are available as single tablet complete PEP regimens: bictegravir/tenofovir alafenamide /emtricitabine (BIC/TAF/FTC; a preferred regimen) and darunavir/cobicistat/tenofovir alafenamide /emtricitabine (DRV/c/TAF/FTC; an alternative regimen). Generic drug forms are available for darunavir (DRV), ritonavir (RTV), tenofovir disoproxil fumarate (TDF)/emtricitabine (FTC), TDF/lamivudine (3TC), and 3TC. Additional prescribing information including other combination products is available in Table 7. Antiviral potency, tolerability, client preferences, cost, and access are all considerations when selecting an nPEP regimen. Some single tablet regimens may be inappropriate for people with organ dysfunction. Healthcare professionals unfamiliar with these medications should use local infectious diseases or other expert consultation resources, or call the National Clinician Consultation Center PEPline [888-448-4911 or https://nccc.ucsf.edu/clinician-consultation/pep-post-exposure-prophylaxis/] or the Perinatal HIV Line [888-448-8765 or https://nccc.ucsf.edu/clinician-consultation/perinatal-hiv-aids/].

†† Regimens within categories are listed in alphabetical order and not according to preference.

§§ The prescribing information for lamivudine recommends dosage adjustment from 300 mg once daily to 150 mg once daily for patients with CrCl 30–49 mL/min. However, the prescribing information for several fixed-dose combination (FDC) products that contain lamivudine recommends no dose adjustment for CrCl 30–49 mL/min. Therefore, no dose adjustment is needed for lamivudine when administered as a standalone tablet or part of a FDC tablet.

*** Tenofovir DF (TDF) 300 mg every 48 hours.

††† Please see manufacturer’s package insert for dosing instructions for individual agents or consult the Guidelines for the Use of Antiretroviral Agents.

### Management of occupational exposures to source patients with HIV and undetectable serum viral loads

1. Consult a provider with expertise in HIV treatment^*^ (see Box 1). **Good Practice Statement,** Existing Recommendation
2. Use shared clinical decision-making^†^ involving the exposed HCP following occupational exposure to a patient with an undetectable serum viral load when deciding whether to forego initiation of PEP or discontinue PEP early (see **Exposure to a Source Patient with HIV Infection and an Undetectable Plasma Viral Load**). **Recommendation,** Moderate Confidence

### Management of occupational exposures in HCP on pre-exposure prophylaxis for HIV

1. Consult a provider with expertise in HIV treatment^*^ (see Box 1). **Good Practice Statement,** Existing Recommendation
2. Use shared clinical decision-making^†^ involving the exposed HCP taking pre-exposure prophylaxis (PrEP) when deciding whether to forego initiation of PEP or discontinue PEP early (see **HIV PEP Regimens for HCP taking Pre-Exposure Prophylaxis for HIV)**. **Good Practice Statement,** Indirect Evidence

### Management of occupational exposures to an unknown source (e.g., needle in sharps disposal container)

1. Consult a provider with expertise in HIV treatment^*^ (see Box 1). **Good Practice Statement,** Existing Recommendation
2. Use shared clinical decision-making^†^ involving the exposed HCP when deciding whether to forego initiation of PEP or discontinue PEP early (see **Exposure to an Unknown Source)**. **Good Practice Statement,** Existing Recommendation
3. Do not test needles or other sharp instruments for HIV. **Good Practice Statement,** Existing Recommendation

### Management of occupational exposures to source patients with drug-resistant HIV

1. If a source patient is known to have drug-resistant HIV:
  - Consult a provider with expertise in HIV treatment^*^ to assist in the selection of a PEP regimen to which the source patient’s virus is likely to be susceptible (see Box 1).
  - Do not delay the initiation of PEP while awaiting expert consultation with a provider with expertise in HIV treatment^*^.
  - Modify the regimen after PEP has been initiated whenever such modifications are deemed appropriate (e.g., if source patient drug resistance information becomes available later).

**Good Practice Statement,** Existing Recommendation

### Laboratory testing of exposed HCP

1. Perform baseline laboratory tests of exposed HCP as soon as possible after exposure, including:
  - A rapid or lab-based fourth-generation HIV Ag/Ab combination immunoassay, and
  - Serum creatinine, aspartate transaminase (AST) and alanine transaminase (ALT).

**Good Practice Statement,** Existing Recommendation

1. Perform additional baseline nucleic acid testing (NAT)^§^ **only** for exposed HCP who have received cabotegravir-based PrEP in the past 12 months, in consultation with a provider with expertise in HIV treatment. (see **HIV PEP Regimens for HCP taking Pre-Exposure Prophylaxis for HIV)**. **Good Practice Statement,** Indirect Evidence
2. Perform interim HIV tests using lab-based HIV Ag/Ab combination immunoassay and NAT at weeks 4–6 post-exposure **only** for exposed HCP who initiated PEP more than 24 hours after a single exposure or who missed any PEP doses. **Good Practice Statement,** Expert Experience
3. Perform final HIV tests of exposed HCP using lab-based HIV Ag/Ab combination immunoassay and NAT at week 12 post-exposure. **Recommendation,** Moderate Confidence
4. Perform follow-up testing of serum creatinine, AST, and ALT only when baseline tests are abnormal or there are clinical indications (e.g., signs or symptoms of kidney or liver injury). **Good Practice Statement,** Expert Experience
5. Consider performing additional laboratory testing on a case-by-case basis when indicated based upon HCP’s specific clinical situation or other medical comorbidities. **Good Practice Statement,** Existing Recommendation
6. Perform laboratory-based HIV tests (e.g., HIV RNA test) for any exposed HCP who has an illness compatible with an acute retroviral syndrome, regardless of the interval since exposure. **Good Practice Statement,** Existing Recommendation

^*^An expert refers to a healthcare professional with expertise in HIV treatment, antiretroviral medication use, and determination of HIV exposure risk.

†Shared clinical decision-making recommendations are intended to be flexible and should be informed by the characteristics, values, and preferences of the individual patient and the clinical discretion of the healthcare provider.

§Nucleic acid tests (NATs) are qualitative tests that detect the presence of HIV RNA (e.g., HIV-1 RNA assay) and quantitative tests that measure the amount of HIV RNA in the plasma (e.g., viral load). Diagnostic HIV NATs (i.e., qualitative) are recommended because they are more likely than viral load tests to detect very low levels of HIV.

## Background

### Definition of HCP and occupational exposure to HIV

The term HCP refers to all paid and unpaid persons serving in healthcare settings who have the potential for direct or indirect exposure to patients or infectious materials, including body substances (e.g., blood, tissue, and specific or blood-contaminated body fluids), contaminated medical supplies, devices, equipment, environmental surfaces, or air.^17^ These HCP may include but are not limited to, emergency medical service personnel, nurses, nursing assistants, physicians, technicians, therapists, phlebotomists, pharmacists, students and trainees, contractual staff not employed by the healthcare facility, and persons (e.g., clerical, dietary, environmental services, laundry, security, maintenance, engineering and facilities management, administrative, billing, and volunteer personnel) not directly involved in patient care but potentially exposed to infectious agents that can be transmitted from HCP and patients.

An exposure that might place HCP at risk for HIV infection is defined as a percutaneous injury (e.g., a needlestick or cut with a sharp object) or contact of mucous membranes or nonintact skin (e.g., exposed skin that is chapped, abraded, or afflicted with dermatitis) with blood, tissue, or other body fluids that are potentially infectious. In addition to blood and visibly bloody body fluids, semen, pre-seminal fluid, vaginal fluids, rectal fluids, and breast milk are also considered potentially infectious.^18^ Although HIV may be transmitted sexually via semen and vaginal secretions, there are no known reports of occupational transmission with these fluids from patients to HCP. The following fluids are also considered potentially infectious: cerebrospinal fluid, synovial fluid, pleural fluid, peritoneal fluid, pericardial fluid, and amniotic fluid. The risk for transmission of HIV infection from these fluids is unknown; the potential risk to HCP from occupational exposures to these fluids has not been assessed by epidemiologic studies in healthcare settings. Feces, nasal secretions, saliva, sputum, sweat, tears, urine, and vomitus are not considered potentially infectious unless they are visibly bloody.^18,19^

Any direct contact (i.e., contact in the absence of personal protective equipment) with concentrated virus in a research laboratory or production facility requires clinical evaluation.^20^ For human bites, clinical evaluation must include the possibility that both the person bitten and the person who inflicted the bite were exposed to blood-borne pathogens. Transmission of HIV infection by this route has been reported rarely, but not after an occupational exposure in healthcare.^21–27^

### Risk for occupational transmission of HIV

Factors associated with risk for occupational transmission of HIV have been described; risks vary with the type and severity of exposure.^19,28,29^ In prospective studies of HCP, the average risk for HIV transmission after a percutaneous exposure to HIV-infected blood has been estimated to be 0.23% (95 % confidence interval [CI], 0.00%–0.46%)^30^ and that after a mucous membrane exposure to be approximately 0.09% (95% CI, 0.006%–0.5%).^31^ Although episodes of HIV transmission after nonintact skin exposure have been documented, the average risk for transmission by this route has not been precisely quantified but is estimated to be less than the risk for mucous membrane exposures. The risk for transmission after occupational exposure to fluids or tissues other than HIVinfected blood also has not been quantified but may be considerably lower than that for blood exposures.^19^

Epidemiologic and laboratory studies suggest that multiple factors might affect the risk of HIV transmission after an occupational exposure.^32^ In a retrospective case-control study of HCP who had percutaneous exposure to HIV, increased risk for HIV infection was associated with exposure to a larger quantity of blood from the source person as indicated by (1) a device (e.g., a needle) visibly contaminated with the patient’s blood, (2) a procedure that involved a needle being placed directly in a vein or artery, or (3) a deep injury. The risk also was increased for exposure to blood from source persons with terminal illness, likely reflecting the higher viral inoculum of HIV in blood late in the clinical course of acquired immunodeficiency syndrome (AIDS). Taken together, these factors suggest a direct inoculum effect (i.e., the larger the viral inoculum, the higher the risk for infection). One laboratory study that demonstrated that more blood is transferred by deeper injuries and hollow-bore needles lends further credence to the observed variation in risk related to inoculum size.^33^

### Timing and duration of PEP

Animal studies have suggested that PEP is most efficacious when begun as soon as possible after the exposure and becomes less effective when started 48 to 72 hours after exposure, supporting that the initiation of PEP after exposure is an urgent matter.^34–38^ Hence, for example, a surgeon who sustains an occupational exposure to HIV while performing a surgical procedure has an urgent reason to, after assuring the safety of the patient, promptly scrub out of the surgical case and seek immediate medical evaluation.

The precise interval after which no benefit is gained from PEP is uncertain for humans. The evidence retrieved by the systematic review is inconclusive because included studies had small sample sizes, low or no events, and a corresponding absence of statistical analyses. The longer the initiation of the PEP regimen is delayed beyond 72 hours, the greater the chances are that the regimen’s potential efficacy will be outweighed by even a small likelihood of its adverse events. The optimal duration of PEP is unknown; however, duration of treatment has been shown to influence success of PEP in animal models.^35^ Because four weeks of PEP appeared protective in in vitro, animal,^34,35,39,40^ and occupational^32^ studies, PEP has historically been administered for a four-week course.

### Antiretroviral agents for PEP

ART regimens from eight classes of drugs are currently available to treat HIV infection.^16^ These include the nucleoside and nucleotide reverse-transcriptase inhibitors (NRTIs), nonnucleoside reverse-transcriptase inhibitors (NNRTIs), protease inhibitors (PIs), integrase strand transfer inhibitors (INSTIs), a fusion inhibitor (FI), chemokine (C-C motif) receptor 5 (CCR5) antagonists (entry inhibitors), post-attachment inhibitors, and capsid inhibitors. Only ART regimens approved by the FDA for treatment of HIV infection are included in these guidelines, although none of these agents has an FDA-approved indication for administration as PEP. The rationale for offering ART regimens as PEP is based on our understanding of the pathogenesis of HIV infection and the plausibility of pharmacologic intervention in this process, studies of the efficacy of antiretroviral chemoprophylaxis in animal models,^34–37^ and epidemiologic data from HIV-exposed HCP^32,41^.

### Antiretroviral agents for PEP: selection, duration, toxicity, and drug interactions

Selection of PEP regimens may be complex due to potential challenges involved in determining whether an exposure constitutes a risk that would warrant PEP, and some providers’ lack of experience with prescribing PEP. The recommended infrastructure and routine practices for providing exposure management services for HCP is detailed in CDC’s Infection Control in HCP guideline, and includes facilitating access to expert consultants who are available 24 hours a day and seven days per week.^15^

A three-drug treatment paradigm has been applied successfully for PEP, with modern ART regimens being better tolerated than agents previously used for PEP in the pre-INSTI era.^16^ The recommended initial regimens (Table 2) have been associated with minimal or tolerable side effects and limited drug-drug interactions. Additionally, the results of the systematic literature review (see Appendix) suggested no difference in effectiveness between dolutegravir- or bictegravir-based ART regimens and raltegravir-based as PEP in nonoccupational populations. Notably, one of the regimens (bictegravir + tenofovir alafenamide + emtricitabine) is available as a single tablet, once-daily option as Biktarvy. While two-drug regimens are used for some patients as HIV treatment, there are no data on their effectiveness as PEP, and their effectiveness for high-risk percutaneous exposures such as may occur in occupational settings is also unknown. The finite four-week duration of PEP also means that the risk for cumulative toxicity of three-drug regimens compared to two-drug regimens is minimal. Additionally, in the theoretical event of an infection occurring despite taking PEP for an occupational exposure, there would presumably be less risk for the development of drug resistance on a three-drug compared to a two-drug regimen.

The preferred initial PEP regimens recommended in this update have low rates of side effects which in the majority of cases are mild in severity; therefore, alternative initial regimen(s) are likely to be needed only infrequently for select cases. Their favorable side effect profile as well as their convenient dosing schedule facilitates both adherence to the regimen and completion of 4 weeks of PEP. A discussion about potential side effects of the agents in the PEP regimen with the recipient can promote adherence to the regimen. Certain adverse events reported for ART agents were reported primarily for persons taking them for prolonged periods of time for treatment of HIV infection, and therefore may not reflect the experience of uninfected persons who take PEP for four weeks. Information regarding potential drug interactions has been published,^16^ and up-to-date information can be found in the University of Liverpool HIV drug interaction tracker (https://www.hiv-druginteractions.org). Additional information is included in manufacturers’ package inserts. Serious adverse side effects can be reported to FDA’s MedWatch program.^42^

There have been no reports of any occupational HIV transmissions when HCP have taken the regimen of raltegravir, tenofovir DF, and emtricitabine, which was the preferred PEP regimen in the 2013 PHS Guidelines. Its exclusion from the preferred and alternative regimens in Table 2 is due to its higher pill burden and the availability of once-daily antiretroviral regimens, rather than any concerns regarding efficacy.

Cabotegravir is co-packaged with rilpivirine (Cabenuva) for the treatment of HIV-1, and is available separately (Apretude) for pre-exposure prophylaxis of HIV. Both cabotegravir and rilpivirine are available as extended-release injectable suspensions administered IM. The systematic literature review did not retrieve studies evaluating cabotegravir-containing regimens prescribed as alternative PEP regimens among any populations. Due to its prolonged suppressive effect on HIV, IM cabotegravir or Cabenuva may substantially delay the time to detection of HIV antibodies and/or viral load for months,^43^ requiring long follow-up testing beyond the final HIV diagnostic test at 12 weeks post-exposure for oral PEP regimens (see **Source Patient HIV Testing**). A recent animal study showed that PEP with long-acting cabotegravir and rilpivirine was partially effective and demonstrated late breakthrough infections, highlighting the limitations of this regimen for PEP.^44^ Accordingly, due to the need for long-term follow-up testing and the lack of clinical data on its safety and effectiveness when used as PEP, cabotegravir/rilpivirine was not included in the list of preferred or alternative regimens.

Nevirapine remains contraindicated due to reports of hepatic failure in patients taking this as PEP.^45^

### Resistance to antiretroviral agents

Information on whether a source patient harbors drug-resistant HIV may be unclear or unavailable at the time of an occupational exposure and when PEP is prescribed. ART resistance is typically suspected in a source patient who experiences clinical progression of disease, a persistently increasing viral load, or a decline in CD4+ T cell count despite therapy and in instances in which a virologic response to therapy fails to occur. However, resistance testing of the source patient’s virus at the time of an exposure to aid in the selection of the initial PEP regimen is impractical, because the results will not be available in time to influence the choice of the initial PEP regimen.

Known or suspected resistance of the source patient’s virus to one or more ART agents that might be included in a PEP regimen raises concerns about reduced PEP efficacy of that regimen.^46^ Drug resistance to all available ART agents has been reported, and cross-resistance within drug classes occurs frequently.^16^ Occupational transmission of drug-resistant HIV strains, despite PEP with combination drug regimens, has been reported.^47–49^

### Antiretroviral drugs during pregnancy or breastfeeding

The risk of HIV transmission poses a threat to both the pregnant HCP as well as their fetus, as the risk of perinatal HIV transmission is markedly increased during acute HIV infection during pregnancy.^50^ Including obstetric providers (e.g., obstetrician, family medicine provider, midwife) of pregnant HCP in post-exposure medical care is also critical for their safety and health. Although the initiation of PEP is not delayed, when pregnant HCP or HCP intending to become pregnant have an occupational exposure or occupational acquisition of an infectious disease, OHS will typically refer exposed HCP to their obstetric provider so that ongoing recommended post-exposure management and counseling can be collaboratively managed.^51^

The potential risks associated with ART exposure for pregnant HCP, fetuses, and infants depend on the timing and duration of exposure as well as the number and type of drugs. Preferred and alternative regimens for use in pregnancy are available in Table 2. Cobicistat-containing regimens are not recommended for use in pregnancy by the HHS Services Panel on Treatment of HIV During Pregnancy and Prevention of Perinatal Transmission due to reduced plasma drug exposure.^12^

Antiretroviral drug levels in breast milk vary among drugs with administration of some drugs resulting in high levels (e.g., lamivudine), while other drugs, such as protease inhibitors and tenofovir disoproxil fumarate (TDF), are associated with only limited penetration into milk.^52,53^ Administration of antiretroviral triple-drug regimens to breastfeeding women with HIV has been shown to decrease the risk of transmission to their infants and infant toxicity has been minimal.^12^ The risk of infant serious adverse events from maternal antiretroviral drug use during breastfeeding is very low.

### HIV post-exposure prophylaxis regimens for HCP with kidney disease

Some ART agents require dose-adjustments or are contraindicated for use in patients with kidney disease. An expert in the use of ART agents can help with the selection and dosing of the most appropriate regimen. See Table 2 for more information.

### HIV post-exposure prophylaxis regimens for HCP taking pre-exposure prophylaxis (PrEP) for HIV

Although pre-exposure prophylaxis (PrEP) is highly effective at preventing HIV transmission through sexual exposures, its effectiveness for prevention of occupationally acquired HIV transmission is unknown.^54^ If occupational health providers are using shared decision-making to determine whether or not occupationally-exposed HCP who are already taking PrEP can forego initiation of PEP or discontinue PEP early, considerations include: (1) recency of initiation of PrEP; (2) adherence to PrEP regimens and any missed PrEP doses; (3) use of intermittent PrEP regimens outside of current CDC PrEP guideline recommendations; (4) exposure to source patients without sustained viral suppression and resistance to PrEP components; and (5) the potential risks and benefits of a 3-drug PEP regimen compared to a 1–2 drug PrEP regimen.

PrEP regimens that contain long-acting cabotegravir have a prolonged elimination half-life.^55^ Although these regimens are highly effective, patients who acquire HIV infection while on them may benefit from diagnostic HIV NAT testing which increases sensitivity (compared to Ag/Ab tests).

### Management of occupational exposure by emergency providers

Many HCP exposures to HIV occur outside of occupational health clinic hours of operation and at sites at which occupational health services are unavailable, with initial exposure management often overseen by emergency physicians or other providers who are not experts in the treatment of HIV infection or the use of ART agents. These providers may not be familiar with either the PHS guidelines for the management of occupational exposures to HIV or the available ART agents and their relative risks and benefits. Previous focus groups conducted among emergency department physicians who had managed occupational exposures to blood and body fluids^56^ identified three challenges in occupational exposure management: evaluation of an unknown source patient or a source patient who refused testing, inexperience in managing occupational HIV exposures, and counseling of exposed HCP in busy emergency departments. Readily-available protocols for the management of occupational exposures to HIV (e.g., disseminated to staff on pocket-sized cards, smartphone app) which provide information on a formal expert consultation mechanism (e.g., the in-house infectious diseases consultant or PEPline), appropriate initial source patient and exposed provider laboratory testing, procedures for counseling of the exposed provider, identifying and having an initial PEP regimen available, and the mechanism for outpatient follow-up, can all facilitate high quality post-exposure management for HCP. The development and use of such protocols is recommended by CDC.^15^

### Source patient HIV testing

Although concerns have been expressed about HIV-negative sources who might be in the so-called window period before seroconversion (i.e., the period between initial HIV infection and the development of detectable HIV antibodies), no such instances of occupational transmission have been reported in the United States to date. Hence, investigation of whether a source patient might be in the window period is not typically performed for determining whether PEP is indicated unless acute retroviral syndrome is clinically suspected. Rapid HIV testing of source patients facilitates timely decision-making regarding the need for administration of PEP after occupational exposures to sources whose HIV status is unknown. FDA-approved rapid tests can produce HIV test results within 30 minutes.^57^ Combination p24 antigen-HIV antibody (Ag/Ab) rapid tests produce both rapid and highly accurate results, and their p24 antigen detection allows improved identification of most infections during the window period.^58^

### Exposure to a source patient with HIV infection and an undetectable plasma viral load

It is uncertain if occupational exposure to a source patient with an undetectable plasma viral load poses a risk of HIV transmission. For HIV-serodiscordant sexual partners, data on the absence of HIV transmission from a partner taking ART with sustained undetectable HIV has underpinned a principle commonly used by HIV clinician experts that undetectable means untransmittable (or “undetectable = untransmittable).”^59^ The systematic literature review retrieved nine studies in both occupationally- and non-occupationally-exposed populations and found no instances of HIV transmission from source patients with undetectable HIV viral loads to exposed HCP, most of whom took PEP courses (see Appendix). A technical report suggested by subject-matter experts did not meet inclusion criteria. It examined 8,292 HCP with occupational exposures from 1997 to 2018 in the United Kingdom^60^; 3,385 of these HCP started PEP, and while source patient viral loads were not reported, it is possible that some proportion of these had undetectable viral loads. The only reported seroconversion involved exposure to a heavily treatment-experienced source patient with a detectable viral load. There have also been no reports of HIV infection through occupational exposure since 2008^61^ (the earliest date searchable electronically) to the CDC’s Cases of Public Health Importance (COPHI) coordinator (unpublished data). If occupational health providers are using shared decision-making to determine whether or not occupationally-exposed HCP to a source patient with an undetectable plasma viral load on ART can forego initiation of PEP or discontinue PEP early, considerations may include: (1) route of inoculation (e.g., percutaneous to deep tissue with large-bore needle); (2) size of inoculum (e.g., large volume of blood); and (3) source patient use of and adherence to ART. The greater the risk of the occupational exposure to the source patient with undetectable viral load is, the greater the potential that the regimen’s adverse effects will be outweighed by its potential effectiveness.

### Exposure to an unknown source

Occupational exposure to an unknown source (e.g., needle in sharps disposal container or laundry) provides challenges in post-exposure management as the risk of exposure to HIV may never be known. There are no approved methods for HIV testing of needles and sharps to provide accurate results. If occupational health providers are using shared decision-making to determine whether or not HCP can forego initiation of PEP or discontinue PEP early in this scenario, considerations may include: (1) severity of exposure (e.g., large volume exposure, exposure with blood), (2) route of inoculation (e.g., percutaneous to deep tissue with large-bore needle), (3) size of inoculum (e.g., large volume of blood), and (4) epidemiologic likelihood of HIV exposure.

## Follow-up of exposed HCP

### Importance of follow-up appointments

Follow-up counseling, post-exposure testing, and medical evaluation are important post-exposure interventions for HCP with occupational exposures to HIV, regardless of whether they take PEP. Careful attention to follow-up evaluation within 72 hours of exposure can (1) provide another (and perhaps less anxiety ridden) opportunity to allow the exposed HCP to ask questions and for the counselor to make certain that the exposed HCP has a clear understanding of the risks for infection and the risks and benefits of PEP, (2) ensure that continued treatment with PEP is indicated, (3) increase adherence to PEP regimens, (4) detect and manage associated symptoms and side effects more effectively, (5) provide an early opportunity for ancillary medications or regimen changes, (6) improve detection of serious adverse effects, and (7) improve the likelihood of follow-up serologic testing for a larger proportion of exposed personnel to detect infection. Closer follow-up may in turn reassure HCP who become anxious after these events.^62,63^ The psychological impact of needlesticks or exposure to blood or body fluid should not be underestimated for HCP.

### Post-exposure testing

Data from laboratory studies suggest that the time from infection to laboratory detection is less than three months following HIV exposure when using fourth-generation tests (see Appendix).^64,65^ One study examining HIV NAT tests from a cohort of plasma specimens from US HIV seroconverters who had not taken PEP found that this time interval was 33 days for the 99^th^ percentile of the cohort.^64^ Accordingly, the rationale for the timepoint of the final post-exposure test is to account for this time period in addition to the duration of PEP course and an interim period of antiretroviral drug washout following completion.

Historically, for people who have been adherent to PEP regimens, interim testing for HIV has not been reported by experts in the field to detect incident HIV seroconversion. However, for instances where PEP was initiated later than 24 hours after a single exposure, or for those who missed any doses of PEP, there may be increased risk for seroconversion, and therefore interim HIV testing and detection may be beneficial for the exposed HCP.

Due to the low toxicity profile of the recommended and alternative regimens as well as the short duration of PEP, it is not anticipated that interim laboratory tests (e.g., serum creatinine, ALT, or AST testing) to detect drug-induced toxicities will be beneficial.

Extended HIV follow-up testing (e.g., for four-six months) for HCP who become infected with HCV after exposure to a source patient who is co-infected with HIV and HCV is no longer used due to the greater sensitivity that widely available HIV antigen/antibody tests have compared to older generation HIV tests.

HIV is a nationally notifiable disease.^66^ Professional societies have offered guidance on management of the HIV-infected HCP.^67^ Reporting of occupationally-acquired HIV infections to the CDC’s COPHI coordinator, which is voluntary, may be done by calling 404-639-2050.

## Reevaluation and update of HIV occupational PEP guidelines

As new ART agents for treatment of HIV infection and additional information concerning early HIV infection and prevention of HIV transmission become available, the interagency PHS working group will assess the need to update these guidelines. Updates will be published periodically as appropriate.

## Expert panel consultants

Elaine Abrams, MD, Columbia University Medical Center; Judith Aberg, MD, FIDSA, FACP, Icahn School of Medicine at Mount Sinai; Hilary Babcock, MD, MPH, Washington University School of Medicine in St. Louis; Mark Russi, MD, MPH, Yale School of Medicine; Susan Eshleman, MD, PhD, Johns Hopkins University School of Medicine; Carolyn Chu, MD, University of California San Francisco School of Medicine.

## OMB peer review panel

Sigal Yawetz, MD, Brigham and Women’s Hospital, Harvard Medical School; Susan Buchbinder, MD, San Francisco Department of Public Health; David J. Weber, MD, MPH, University of North Carolina Medical Center.

## Supporting information

Kofman et al. supplementary material Kofman et al. supplementary material

## Supplementary material

To view supplementary material for this article, please visit https://doi.org/10.1017/ice.2025.10254.
