# Supplementary material for: 2025 US Public Health Service Guidelines for the Management of Occupational Exposures to Human Immunodeficiency Virus and Recommendations for Post-exposure Prophylaxis in Healthcare Settings
Source: Infect Control Hosp Epidemiol. 2025 Sep 15;46(9):863–73. doi: 10.1017/ice.2025.10254 (PMC12616222; doi:10.1017/ice.2025.10254)
Supplement: Kofman et al. supplementary material [file S0899823X25102547sup001.docx]

# 2025 US Public Health Service Guidelines for the Management of Occupational Exposures to Human Immunodeficiency Virus and Recommendations for Postexposure Prophylaxis in Healthcare Settings: Appendix

Contents

2025 [US Public Health Service Guidelines for the Management of Occupational Exposures to Human Immunodeficiency Virus and Recommendations for Postexposure Prophylaxis in Healthcare Settings: Appendix 1](#_Toc202967020)

[**A. Methods** 5](#_Toc202967021)

[**A.1. Key Question and Literature Search Development** 5](#_Toc202967022)

[**A.2. Study Selection** 7](#_Toc202967023)

[**A.3. Data Extraction, Appraisal, and Evidence Evaluation** 10](#_Toc202967024)

[**A.4. Formulating Recommendations** 10](#_Toc202967025)

[**B. Summary of Recommendations and Rationale** 13](#_Toc202967026)

[**C. KQ1 Antiretroviral Therapy Dolutegravir and/or Bictegravir-based ART Regimens** 17](#_Toc202967027)

[**C.1. KQ1 Recommendation and Justifications** 17](#_Toc202967028)

[**C.2. KQ1 Summary of Findings and Evidence Evaluation** 18](#_Toc202967029)

[**C.3 KQ1 Narrative Summary of Evidence** 18](#_Toc202967030)

[**D. KQ2 Antiretroviral Therapy IM-administered Cabotegravir-based ART Regimens** 21](#_Toc202967031)

[**D.1. KQ2 Recommendation and Justifications** 21](#_Toc202967032)

[**D.2. KQ2 Summary of Findings and Evidence Evaluation** 21](#_Toc202967033)

[**D.3. KQ2 Narrative Summary of Evidence** 21](#_Toc202967034)

[**E. KQ3 Time Interval Between Exposure and PEP Administration** 23](#_Toc202967035)

[**E.1. KQ3 Recommendation and Justifications** 23](#_Toc202967036)

[**E.2. KQ3 Summary of Findings and Evidence Evaluation** 24](#_Toc202967037)

[**E.3. KQ3 Narrative Summary of Evidence** 24](#_Toc202967038)

[**F. KQ4 PEP Indications** 26](#_Toc202967039)

[**F.1. KQ4 Recommendation and Justifications** 26](#_Toc202967040)

[**F.2. KQ4 Summary of Findings and Evidence Evaluation** 26](#_Toc202967041)

[**F.3. KQ4 Narrative Summary of Evidence** 26](#_Toc202967042)

[**G. KQ5 Fourth Generation HIV Tests** 29](#_Toc202967043)

[**G.1. KQ5 Recommendation and Justifications** 29](#_Toc202967044)

[**G.2. KQ5 Summary of Findings and Evidence Evaluation** 29](#_Toc202967045)

[**G.3. KQ5 Narrative Summary of Evidence** 30](#_Toc202967046)

[**H. Management and Testing for HCP on PrEP** 33](#_Toc202967047)

[**H.1 Management of Occupational Exposures in HCP on PrEP: Recommendation and Justifications** 33](#_Toc202967048)

[**H.2 Repeat Testing for Exposed HCP on PrEP: Recommendation and Justifications** 34](#_Toc202967049)

[**I. Included Evidence** 36](#_Toc202967050)

[**I.1 Extracted Evidence from Individual Studies** 36](#_Toc202967051)

[**I.2. Assessment of Bias for Individual Studies** 64](#_Toc202967052)

[**I.3. Summary of Expert Evidence** 64](#_Toc202967053)

[**J. Search Strategies** 66](#_Toc202967054)

[**K. All References in the Main Document and Appendix** 70](#_Toc202967055)

**Table of Tables**

[**Table S1a.** Recommendation categorization framework 10](#_Toc202967056)

[**Table S1b.** Definitions for confidence in the evidence 11](#_Toc202967057)

[**Table S2.** Recommendation strength & rationale for all recommendations* 12](#_Toc202967058)

[**Table S3.** Recommendation justification^3^ for dolutegravir and/or bictegravir-based ART regimens 16](#_Toc202967059)

[**Table S4.** GRADE Table: Dolutegravir-based antiretroviral regimen compared to raltegravir plus emtricitabine/tenofovir 17](#_Toc202967060)

[**Table S5.** Recommendation justification^3^ for IM-administered cabotegravir-based ART regimens 20](#_Toc202967061)

[**Table S6**. Recommendation justification^3^ for the time interval between exposure and PEP administration 22](#_Toc202967062)

[**Table S7.** GRADE Table: HIV seroconversion when Initiating PEP before or after 72 hours of HIV exposure 23](#_Toc202967063)

[**Table S8.** Recommendation Jjustification^3^ for IM-administered cabotegravir-based ART regimens 25](#_Toc202967064)

[**Table S9.** Recommendation justification^3^ for fourth generation HIV tests 28](#_Toc202967065)

[**Table S10.** GRADE Table: Duration to positive test/ detection when using fourth generation HIV tests 28](#_Toc202967066)

[**Table S11**. Recommendation fustification^3^ for shared decision-making involving the exposed HCP taking PrEP 32](#_Toc202967067)

[**Table S12**. Recommendation justification^3^ for additional baseline qualitative NAT test for exposed HCP who have received cabotegravir-based PrEP in the last 12 months. 33](#_Toc202967068)

[**Table S13.** GRADE Table: HIV seroconversion following exposure to source patients with undetectable HIV viral load 34](#_Toc202967069)

[**Table S14**. Select extracted data from studies reporting antiretroviral therapy outcomes of interest 35](#_Toc202967070)

[**Table S15.** Summary of expert experience informing the development of Good Practice Statements. 64](#_Toc202967071)

[**Table S16.** Literature search strategies and results by database 65](#_Toc202967072)

# **A. Methods**

This document is an update of the *2013 U.S. Public Health Service Guidelines for the Management of Occupational Exposures to HIV and Recommendations for Postexposure^1,2^* (hereafter referred to as “*2013 PHS Guidelines*”)*.* To conduct this update, the Centers for Disease Control and Prevention (CDC) assembled representatives from the U.S. Food and Drug Administration (FDA), the National Institutes of Health (NIH), the Health Resources and Services Administration (HRSA), and CDC to collaborate on this update, with discrete input from occupational post-exposure prophylaxis (PEP) subject matter experts from the field. As in the main guideline document, an occupational exposure to HIV is defined as a percutaneous injury (e.g., a needlestick or cut with a sharp object) or contact of mucous membranes or non­intact skin (e.g., exposed skin that is chapped, abraded, or afflicted with dermatitis) with blood, tissue, or other body fluids that are potentially infectious. In addition to blood and visibly bloody body fluids, semen, pre-seminal fluid, vaginal fluids, rectal fluids, and breast milk are also considered potentially infectious.^18^

CDC experts reviewed the 2013 PHS Guidelines*^1,2^* to identify recommendations that did not require a change in meaning, existing 2013 recommendations that required clarification, and new topics that have emerged since the last publication. Recommendations that did not require a change were adopted and are indicated in Table S2.

## **A.1. Key Question and Literature Search Development**

New topics identified by CDC and approved by the expert panel included antiretroviral therapy, timing of PEP, indications for PEP, and testing. CDC experts and methodologists developed a list of Key Questions using the PICO framework^68^ (population, intervention, comparator), which were vetted with federal partners and external experts. The Key Questions used to guide the systematic literature review are:

1. What is the balance of benefits and risks of prescribing dolutegravir and/or bictegravir-containing regimens compared to raltegravir plus emtricitabine/tenofovir for use as the preferred PEP among healthcare personnel with an occupational exposure to HIV?
2. What is the balance of benefits and risks of prescribing intramuscular (IM)-administered cabotegravir-based ART regimens compared to the standard of care of use as an alternative PEP regimen among healthcare personnel with an occupational exposure to HIV?
3. What is the interval following an occupational HIV exposure after which no benefit is gained by administering PEP to healthcare personnel?
4. What is the risk of a patient with an undetectable HIV viral load transmitting to HCP following an occupational percutaneous and/or mucous membrane exposure?
5. What is the window of time until HIV antibodies can be detected using a HIV Ag/ab test, among healthcare personnel with an occupational exposure?

A CDC information scientist (J.T.) developed search strategies from the list of Key Questions. The concurrent update to the non-occupational PEP Guidelines resulted in a strict focus on literature related to occupational exposures for these guidelines, and therefore the literature search was not tailored to identify studies reporting on non-occupational populations. The information scientist performed these searches in MEDLINE, EMBASE, Global Health (OVID), Cochrane Library, Nursing and Allied Health Database (ProQuest), and Scopus from the start of each database to June 15, 2023. Subject matter experts were asked to supplement the literature search results by recommending relevant references published since June 2023.

## **A.2. Study Selection**

Titles and abstracts from references were uploaded into Covidence systematic review software (Veritas Health Innovation, Melbourne, Australia. Available at [www.covidence.org](http://www.covidence.org)), and screened by dual review (D.O.S., C.N.S., or E.C.S.). Full-text articles were retrieved if they were relevant to one or more Key Questions.

Per protocol criteria for excluding studies from the literature review include:

1. No full-text available
2. Not written in English
3. Not humans
4. Not primary research
5. Conference abstract or poster
6. No exposures of interest
7. No outcomes of interest
8. Studies reporting on data collected prior to 2010, regardless of publication year, because fourth generation Ag/ab tests were approved by the FDA in 2010

The full texts of selected articles and the relevant references of selected systematic reviews were then screened by two independent reviewers, and disagreements were resolved by discussion (D.O.S., C.N.S., or E.C.S.). Importantly, while the search strategy was tailored to studies reporting on occupational exposures, some non-occupational studies were identified in this literature search, and these did not meet exclusion criteria. After the full-text screening was complete, a bibliography of the articles selected for inclusion was vetted with subject matter experts, who suggested additional studies for Key Questions where retrieved evidence was insufficient to formulate an answer. Additional studies were screened using inclusion criteria that were broadened to include cross sectional and modeling studies, which the original search was not crafted to capture. Once the exclusion criteria were broadened, Key Question five was iteratively refined to account for the new evidence. The original Key Question was: When using a fourth generation HIV Ag/ab test, what is the risk of a breakthrough infection after 3 months post occupational HIV exposure among healthcare personnel? The new Key Question was: What is the window of time until HIV antibodies can be detected using a fourth generation HIV Ag/ab test, among healthcare personnel with an occupational exposure?

The results of the study selection process are depicted in *Figure S1.*

**Figure S1.** Results of the Study Selection Process

5,980 titles/ abstracts screened

4,939 studies excluded

1,041 full-text articles reviewed

1,019 studies excluded

- 590 No intervention of interest
- 181 Study type
- 123 Not written in English
- 104 No outcomes of interest
- 13 No full-text available
- 8 Duplicates

1,269 duplicates removed

7,249 studies identified

- 7,246 via literature searches
- 2 via SMEs
- 1 via reference mining

22 studies included*

- 4 relevant to KQ 1
- 5 relevant to KQ 3
- 9 relevant to KQ 4
- 4 relevant to KQ 5

* Some studies included in more than one KQ

## **A.3. Data Extraction, Appraisal, and Evidence Evaluation**

Methodologic data and results of clinically relevant outcomes from the studies meeting inclusion criteria were extracted into standardized evidence tables. Data and analyses were extracted as presented in the studies. For the purposes of this review, statistical significance was defined as *p* ≤ 0.05. Potential biases in each study were assessed using a tool developed by the Division of Healthcare Quality Promotion at the Centers for Disease Control and Prevention, and biases of concern were recorded in the evidence tables. [Figure S2](#_I.2._Assessment_of) includes the questions used to assess the threats to internal validity.

For Key Questions where evidence was retrieved, the body of evidence was narratively synthesized for each outcome within each Key Question. The strength, direction, consistency, and directness were assessed for each outcome using the GRADE (Grading of Recommendations, Assessment, Development, and Evaluations) approach.^4^

## **A.4. Formulating Recommendations**

Recommendation categories and their definitions with rationale, are found in Table S1a. Evidence-based recommendations were developed using HICPAC evidence to decision frameworks and categorized as Recommendations or Conditional Recommendations^3^. Recommendations that were not informed by systematic literature reviews were categorized as Good Practice Statements if they met the five criteria identified by GRADE, ^5^ and where relevant, supporting rationale is identified in Table S2. For Good Practice Statements where expert experience informed recommendation update or development, the experience of workgroup and expert panel members was collected and summarized as has been done previously.^5,69^ The summary of expert experience is found in Section E.3. All recommendations, their category, rationale, and supporting notes are found in Table S2.

All draft recommendations were shared with the non-occupational HIV post-exposure prophylaxis working group to harmonize recommendations between guidelines where applicable.^3^

**Table S1a.** Recommendation categorization framework

| **Category** | **Criteria/ Rationale** |
| --- | --- |
| **All Statements** | The statement is:   - clear and actionable, and - the message is necessary with regard to actual healthcare practice. |
| **Statements developed using systematic review evidence** | |
| **Recommendation^3^** | A new statement supported by **High or moderate confidence** in systematic review evidence **indicating** benefit or harm |
| **Conditional Recommendation^3^** | A new statement supported by **Low confidence** in systematic review evidence **indicating** benefit or harm |
|  | A new statement supported by **High or moderate confidence** in systematic review evidence **suggesting** benefit or harm |
| **Statements developed using alternate sources of evidence** | |
| **Good Practice Statement^5^** | Good Practice Statements (GPS) are developed when:   - Implementing the GPS results in a large net-positive consequences. - Collecting and summarizing the evidence is a poor use of a guideline panel’s limited time, energy or resources. |
|  | The rationale for a GPS may be that it is   - An **Existing CDC Recommendation** that does not require a change in meaning or update at this time, or a new statement supported by   - high or moderate confidence in a well-documented rationale connecting **Indirect Evidence** from animal, pharmacokinetic mechanism of action, or basic science studies, or   - supported by systematically collected **Expert Experience^5^** for instances when evidence collection would be a poor use of the panel’s time and resources. |

**Table S1b.** Definitions for confidence in the evidence

| Confidence Level | Definition^3,4^ |
| --- | --- |
| High | The true effect is very likely to be close to that of the estimate of the effect. |
| Moderate | The true effect is likely to be close to the estimate of the effect, but there is a possibility that it is substantially different. |
| Low | The true effect may be substantially different from the estimate of the effect. |

Draft recommendations were presented to HICPAC in August 2023 during a public meeting, at which there were no suggested edits or recommendations from either the committee or the public.^14^ In October 2024, additional peer review was solicited from experts in the field of HIV and infection prevention, in compliance with requirements of the U.S. Office of Management and Budget (OMB) for influential scientific assessments. Names, credentials, and affiliations of the OMB peer review panel are listed in the “OMB Peer Review Panel” section. Feedback was incorporated into the finalized guidelines.

# **B.** **Summary of Recommendations and Rationale**

**Table S2.** Recommendation strength & rationale for all recommendations*

| **Recommendation (Category, Rationale if not GPS, ER)** | **Notes** |
| --- | --- |
| **Management of HCP with an Occupational Exposure** |  |
| 1. Educate HCP to report occupational exposures to blood and body fluids as soon as possible to occupational health services. | 2013 PHS Guidelines:  Page 1, Abstract |
| 1. Initiate PEP as soon as possible, up to 72 hours following the occupational exposure to HIV. | Page 1, Abstract 2013  Page 7, Timing and Duration, 2013 |
| 1. When considering initiation of PEP after 72 hours following occupational exposures thought to represent a high risk of transmission, consult a provider who has expertise in HIV treatment | 2013 PHS Guidelines:  Page 8, Box 1 |
| 1. Prescribe PEP regimens for a duration of 28 days | 2013 PHS Guidelines:  Page 7, Timing and Duration |
| 1. Determine the HIV status of the source patient whenever possible to guide appropriate use of PEP | 2013 PHS Guidelines:  Page 1, Abstract |
| 1. Do not delay administration of PEP while waiting for information regarding the source patient’s HIV status. | 2013 PHS Guidelines:  Page 2, Introduction |
| 1. Discontinue PEP and HIV follow-up testing if the source patient is determined to be HIV negative | 2013 PHS Guidelines:  Page 6, HIV PEP |
| 1. Provide counseling to exposed HCP in accordance with CDC recommendations for HCP with occupational exposures^51^, and including the: | 2013 PHS Guidelines:  Page 9, Box 2 |
| - 1. Importance of adherence to PEP; | 2013 PHS Guidelines:  Page 9, Box 2 |
| - 1. Use of precautions (i.e., use of barrier contraception, avoidance of blood or tissue donations) to prevent secondary transmission until the final HIV test post-exposure (see Laboratory Testing of Exposed HCP); | 2013 PHS Guidelines:  Page 9, Box 2 |
| - 1. Drugs (e.g. prescription/nonprescription drugs, nutritional ¬supplements) that should not be taken with PEP or require dose or administration adjustments, side effects of prescribed PEP, and measures (including pharmacologic interventions) that may assist in minimizing side effects; and | 2013 PHS Guidelines:  Page 9, Box 2 |
| - 1. Importance of clinical evaluation if any acute symptoms (i.e., side effects of prescribed PEP, symptoms of acute HIV) develop prior to the final HIV test post-exposure (see Laboratory Testing of Exposed HCP). (Good Practice Statement, Existing Recommendation) | 2013 PHS Guidelines:  Page 9, Box 2 |
| 1. Re-evaluate exposed HCP within 72 hours after occupational exposure to assess for further counseling needs and PEP tolerability. | 2013 PHS Guidelines:  Page 6, HIV PEP |
| 1. Consult a provider who has expertise in HIV treatment for HCP unable to take the initial PEP regimen due to intolerance or toxicity (see Box 1). | 2013 PHS Guidelines:  Page 8, Box 1 |
| 1. Refer HCP determined to have HIV infection to a provider who has expertise in HIV treatment. | 2013 PHS Guidelines: Page 9, Postexposure Testing |
| **Management of Pregnant and Breastfeeding HCP with an Occupational Exposure to HIV** |  |
| 1. Consult a provider who has expertise in HIV treatment* (see Box 1). | 2013 PHS Guidelines: Page 8, Box 1 |
| 1. Provide post-exposure management to pregnant or breastfeeding HCP following occupational exposure based on the same considerations that apply to any HCP following occupational exposure to HIV (see HCP with an Occupational Exposure to HIV). | 2013 PHS Guidelines: Page 5, Antiretroviral Drugs During Pregnancy and Lactation |
| 1. Counsel pregnant HCP regarding potential risks and benefits of selected PEP agents for the fetus. | 2013 PHS Guidelines: Page 5, Antiretroviral Drugs During Pregnancy and Lactation |
| 1. Counsel breastfeeding HCP regarding: | 2013 PHS Guidelines: Page 5, Antiretroviral Drugs During Pregnancy and Lactation |
| 1. The high risk of HIV transmission through breast milk should acute HIV infection occur; and | 2013 PHS Guidelines: Page 5, Antiretroviral Drugs During Pregnancy and Lactation |
| 1. The risks and benefits of continuing breastfeeding while taking PEP and being monitored for HIV seroconversion; or interrupting breastfeeding and discarding breast milk until the final HIV test post-exposure (see Laboratory Testing of Exposed HCP) to eliminate risk of HIV transmission to the infant. | 2013 PHS Guidelines: Page 5, Antiretroviral Drugs During Pregnancy and Lactation |
| **Selection of Agents for HIV PEP** |  |
| 1. Consult Table 1 (Main Document) for initial preferred and alternative PEP regimens for HCP following occupational exposure to HIV. **(Recommendation, Moderate)** | GRADED Systematic Review: Appendix Section D.1  2013 PHS Guidelines:  Page 11, Table A1 |
| **Management of Occupational Exposures to Source Patients with HIV and Undetectable Serum Viral Loads** |  |
| 1. Consult a provider who has expertise in HIV treatment* (see Box 1) | 2013 PHS Guidelines:  Page 9, Box 2 |
| 1. Use shared decision-making involving the exposed HCP following occupational exposure to a patient with an undetectable serum viral load when considering whether to forego initiation of PEP or discontinue PEP early. **(Recommendation, Moderate)** | GRADED Systematic Review:  Appendix Section D.4 |
| **Management of Occupational Exposures in HCP on Pre-Exposure Prophylaxis for HIV** |  |
| 1. Consult a provider who has expertise in HIV treatment. (see Box 1) | 2013 PHS Guidelines: Page 9, Box 2 |
| 1. Use shared decision-making involving the exposed HCP taking PrEP when deciding whether to forego initiation of PEP or discontinue PEP early. **(Good Practice Statement, Indirect Evidence)** | Pharmacokinetic Mechanism of Action^70^ |
| **Management of Occupational Exposures to an Unknown Source (e.g., needle in sharps disposal container)** |  |
| 1. Consult a provider who has expertise in HIV treatment* (see Box 1). | 2013 PHS Guidelines: Page 9, Box 2 |
| 1. Use shared decision-making involving the exposed HCP to consider the severity of exposure and epidemiological likelihood of HIV exposure when deciding whether to forego initiation of PEP or discontinue PEP early. | 2013 PHS Guidelines: Page 8, Box 1 |
| **Management of Occupational Exposures to Source Patients with Drug-Resistant HIV** |  |
| 1. If a source patient is known to have drug-resistant HIV: |  |
| 1. Consult a provider who has expertise in HIV treatment* to assist in the selection of a PEP regimen to which the source patient’s virus is likely to be susceptible (see Box 1). | 2013 PHS Guidelines: Page 9, Box 2 |
| 1. Do not delay the initiation of PEP while awaiting expert consultation with a provider who has expertise in HIV treatment*. | 2013 PHS Guidelines: Page 8, Box 1 |
| 1. Modify the regimen after PEP has been initiated whenever such modifications are deemed appropriate (e.g., if source patient drug resistance information becomes available later). | 2013 PHS Guidelines: Page 8, Box 1 |
| **Laboratory Testing of Exposed HCP** |  |
| 1. Perform baseline laboratory tests of exposed HCP as soon as possible after exposure, including: | 2013 PHS Guidelines: Page 1, Abstract and Page 9, Box 2 |
| 1. A rapid or lab-based fourth generation HIV Ag/Ab combination immunoassay, and | 2013 PHS Guidelines: Page 9, Box 2 |
| 1. Serum creatinine, aspartate transaminase (AST) and alanine transaminase (ALT) | 2013 PHS Guidelines: Page 9, Box 2 |
| 1. Perform an additional baseline qualitative nucleic acid test (NAT) **only** for exposed HCP who have received cabotegravir-based PrEP in the past 12 months. **(Good Practice Statement, Indirect Evidence)** | Pharmacokinetic mechanism of action^55^ |
| 1. Perform interim HIV tests using lab-based HIV Ag/Ab combination immunoassay and qualitative nucleic acid test (NAT) at weeks 4-6 post-exposure **only** for exposed HCP who initiated PEP more than 24 hours after a single exposure, or who missed any PEP doses. | 2013 PHS Guidelines: Page 9, Box 2, language clarified to reflect current standard of care as informed by the experience of the expert panel. |
| 1. Perform final HIV tests using lab-based HIV Ag/Ab combination immunoassay and qualitative nucleic acid test (NAT) at week 12 post-exposure of exposed HCP. (Recommendation, Moderate) | GRADED Systematic Review: Appendix Section D.5 |
| 1. Perform follow-up testing of serum creatinine, AST, and ALT only when baseline tests are abnormal or there are clinical indications (e.g., signs or symptoms concerning for kidney or liver injury) | 2013 PHS Guidelines: Box 2, language clarified to reflect current standard of care as informed by the experience of the expert panel. |
| 1. Consider performing additional laboratory testing on a case-by-case basis when indicated based upon HCP’s specific clinical situation or other medical comorbidities. | 2013 PHS Guidelines: Box 2, language modified to clarify the intent. |
| 1. Perform laboratory-based HIV tests for any exposed HCP who has an illness compatible with an acute retroviral syndrome, regardless of the interval since exposure. | 2013 PHS Guidelines: Page 10, first paragraph |

* All Recommendations are Categorized as Good Practice Statements, with a Rationale of “existing recommendation”, unless otherwise noted.

# **C. KQ1 Antiretroviral Therapy Dolutegravir and/or Bictegravir-based ART Regimens**

## **C.1. KQ1 Recommendation and Justifications**

KQ 1 What is the balance of benefits and harms of prescribing dolutegravir and/or bictegravir-based ART regimens (i.e., DTG-3TC-ABC, DTG-3TC, or BIC-TAF-3TC) compared to raltegravir plus emtricitabine/tenofovir for use as the preferred PEP among healthcare personnel with an occupational exposure to HIV?

- The evidence supports addition of dolutegravir- and bictegravir-based ART regimens to the list of preferred PEP regimens. **Recommendation**, Moderate Evidence^71-75^

**Table S3.** Recommendation justification^3^ for dolutegravir and/or bictegravir-based ART regimens

| **Component** | **Justification** |
| --- | --- |
| Supporting evidence | Four cohort studies^71-74^ and one cross-sectional study^75^ |
| Level of confidence in evidence | Moderate: Despite concerns over small sample sizes or numbers of studies and unclear compliance with PEP, it is not anticipated that these findings will change in light of increased use of PrEP among non-occupational populations and the concomitant reduction in cases. |
| Benefits | No difference in HIV seroconversion between dolutegravir- or bictegravir-based ART regimens; and a decreased occurrence of adverse events resulting in an increased adherence to PEP with dolutegravir- and bictegravir-based ART regimens. |
| Risks and harms | No increase in harms or risks with the use of dolutegravir-based ART regimens. |
| Resource use | Dolutegravir- or bictegravir-based ART regimens may be associated with increased financial resource use; however this data was not retrieved from the evidence. |
| Benefit-Harm Assessment | Benefits outweigh harms |
| Value Judgements | Values include reducing patient harm, reducing pill burden, reducing side effects, and prioritizing adherence. |
| Intentional Vagueness | There is no prioritization of recommended regimens. |
| Exceptions | None |

## **C.2. KQ1 Summary of Findings and Evidence Evaluation**

**Table S4.** GRADE Table: Dolutegravir-based antiretroviral regimen compared to raltegravir plus emtricitabine/tenofovir

| **Outcome** | **Summary** | **No of studies (population)** | **Confidence** |
| --- | --- | --- | --- |
| HIV Seroconversion | Evidence indicates no difference in the effectiveness of dolutegravir-based ART regimens compared to raltegravir plus emtricitabine/tenofovir in preventing HIV seroconversion in non-occupational populations. | N = 2228 non-occupational participants  3 Cohort^71-73^  1 Cross-sectional^75^ | Moderate^[[1]](#footnote-1)^ |
| Adherence | Data indicates there is no difference in adherence between the two regimens. | N = 2209 non-occupational participants  2 Cohort^71,73^  1 Cross-sectional^75^ | Moderate^[[2]](#footnote-2)^ |
| Adverse Events | Limited data from and one cross-sectional study^11, 12^ suggests there is a decrease in adverse events with dolutegravir or bictegravir-based ART regimens compared to raltegravir plus emtricitabine/tenofovir. | N = 639 non-occupational participants  1 cohort^73^  1cross-sectional^75^ | Moderate^[[3]](#footnote-3)^ |

## **C.3 KQ1 Narrative Summary of Evidence**

The literature search did not retrieve any studies examining dolutegravir and/or bictegravir-based ART regimens compared to raltegravir plus emtricitabine/tenofovir regimens administered post-exposure in healthcare personnel (HCP). However, the literature search retrieved four cohort studies^71-74^ and one cross-sectional study^75^ reporting on the administration of several different dolutegravir-based^71-73,75^ and one bictegravir-based^74^ PEP ART regimens in non-occupational populations. These populations included men who have sex with men in China,^73^ people with a high-risk exposure visiting a community health center specializing in HIV in France^71^ and the United States,^74^ adults who had previously used an internet medical platform to access PEP in China,^75^ and patients seen by a sexual assault and partner abuse care program in Canada.^72^

Evidence from three cohort studies^71-73^ and one cross-sectional study^75^ indicate no difference in the effectiveness of dolutegravir -based ART regimens compared to raltegravir plus emtricitabine/tenofovir in preventing HIV seroconversion. Two studies were small^72,73^ and importantly, no seroconversions were reported for participants receiving and completing either regimen in any study.^71-73,75^ In one study,^75^ 9.1% of participants reported continued engagement in high-risk sexual behaviors while using PEP. Confidence in the results of one cohort^71^ are reduced due to a low follow up testing rate of 31%. As these studies were cohort or cross-sectional studies, they were not conducted to directly compare these regimens.^71-73,75^ One important consideration is that this data is less likely to change due to the increasing uptake of PrEP.

One cohort^73^ and one cross sectional^75^ study reported a high proportion of treatment completion regardless of regimen, and one cohort^71^ reported a lower proportion of PEP completion among those who used dolutegravir-based regimens, however this was not found to be significant in an analysis of 12 PEP regimens.

Only one small cohort^73^ and one cross-sectional^75^ study reported adverse events by regimen type, and data suggested no serious adverse events occurred with either regimen, and there was a decrease in mild adverse events among the dolutegravir-based PEP group (e.g., mild gastrointestinal issues, neurological disorders, musculoskeletal pain, fatigue, nausea/vomiting, headaches, dizziness/lightheadedness, and myalgia/arthralgia). Both studies relied on self-reported data.

Evidence from only one phase IV trial^74^ of non-occupational participants is insufficient to assess the balance of benefits and harms for the use of bictegravir-based ART regimens compared to raltegravir plus emtricitabine/tenofovir as the preferred PEP among healthcare personnel with an occupational exposure to HIV. This cohort study reported none of the 152 non-occupational participants who received either bictegravir-based ART or raltegravir-based ART were HIV-antibody positive at 4 weeks or 3 months. The bictegravir-based regimen was less likely to be associated with any adverse event than the raltegravir-based regimen, and as a result there were almost two times as many people who completed the bictegravir-based regimen as prescribed. While this data is emerging, the decrease in adverse events combined with the reduced pill burden and the increase in compliance warrant addition of this regimen as a preferred PEP.

# **D. KQ2 Antiretroviral Therapy IM-administered Cabotegravir-based ART Regimens**

## **D.1. KQ2 Recommendation and Justifications**

KQ 2 What is the balance of benefits and harms of prescribing intramuscular (IM)-administered cabotegravir-based ART regimens (i.e., CAB-RPV) compared to the standard of care for use as an alternative PEP regimen among healthcare personnel with an occupational exposure to HIV?

- Evidence is insufficient to formulate a recommendation for use of IM-administered cabotegravir-based ART regimens (i.e., CAB-RPV) as PEP. **No recommendation**

**Table S5.** Recommendation justification^3^ for IM-administered cabotegravir-based ART regimens

| **Component** | **Justification** |
| --- | --- |
| Supporting evidence | No studies were retrieved by the literature search. |
| Level of confidence in evidence | None: Findings may change as new evidence is published. |
| Benefits | No comparative data on seroconversion. |
| Risks and harms | No retrieved evidence, however, increased adherence to PEP is likely with a less frequent intramuscular regimen. |
| Resource use | No retrieved evidence on resource use. |
| Benefit-Harm Assessment | Insufficient data exist to develop a benefit-harm assessment. |
| Value Judgements | Values include reducing patient harm, reduction of adverse events, and prioritizing adherence. |
| Intentional Vagueness | None |
| Exceptions | None |

## **D.2. KQ2 Summary of Findings and Evidence Evaluation**

No evidence was retrieved by the literature search that could be summarized or evaluated.

## **D.3. KQ2 Narrative Summary of Evidence**

KQ 2: What is the balance of benefits and harms of prescribing IM-administered cabotegravir-based ART regimens (i.e., CAB-RPV) compared to the standard of care for use as an alternative PEP regimen among healthcare personnel with an occupational exposure to HIV?

The literature search didn’t retrieve any studies examining the prescription of IM-administered cabotegravir-based ART regimens (i.e., CAB-RPV) compared to the standard of care for use as an alternative PEP regimen among any population, healthcare personnel with an occupational exposure to HIV or non-occupational populations with any exposure to HIV.

# **E. KQ3 Time Interval Between Exposure and PEP Administration**

## **E.1. KQ3 Recommendation and Justifications**

KQ 3 What is the interval following an occupational HIV exposure after which no benefit is gained by administering PEP to healthcare personnel?

- The evidence is insufficient to change the current standard of care of administration in the first 72 hours.
- The Good Practice Statement remains: Initiate PEP as soon as possible, up to 72 hours following the occupational exposure to HIV. **Good Practice Statement, Indirect Evidence.^35-37,76^**

**Table S6**. Recommendation justification^3^ for the time interval between exposure and PEP administration

| **Component** | **Justification** |
| --- | --- |
| Supporting evidence | Four cohort studies^77-80^ and one cross-sectional study^75^ |
| Level of confidence in evidence | Moderate: Concerns exist over small numbers of events, confounding by compliance and additional exposures, however the findings are unlikely to change. |
| Benefits | No difference in HIV seroconversion before or after 72 hours from the exposure. |
| Risks and harms | No data was retrieved on harms. |
| Resource use | No retrieved evidence on resource use. |
| Benefit-Harm Assessment | Insufficient harms data results in an inability to develop a benefit-harm assessment. |
| Value Judgements | Values include consistency in messaging and recommendations across occupational and non-occupational populations, reducing patient harm, and prioritizing adherence. |
| Intentional Vagueness | None |
| Exceptions | None |

## **E.2. KQ3 Summary of Findings and Evidence Evaluation**

**Table S7.** GRADE Table: HIV seroconversion when Initiating PEP before or after 72 hours of HIV exposure

| **Outcome** | **Summary** | **Studies** | **Confidence** |
| --- | --- | --- | --- |
| HIV Seroconversion | Limited evidence in occupational and non-occupational exposures suggests cases of seroconversion are rare when PEP is started after 72 hours. | N = 8 occupational exposures  N = 1569 non-occupational exposures  1 case series^77^  3 Cohort studies^78-80^  1 Cross-sectional study^75^ | Very Low^[[4]](#footnote-4)^ |

## **E.3. KQ3 Narrative Summary of Evidence**

The literature search retrieved only one study reporting the time interval between occupational exposure and first dose among healthcare personnel exposed between 1981 and 2001.^77^ In this study, only one of the eight reported cases of HIV or AIDS that occurred following occupational exposure started their first PEP dose >72 hours post-exposure (at 192 hours). However, the literature search retrieved three cohort studies^78-80^ and one cross-sectional study^75^ reporting outcomes for the interval between a non-occupational HIV exposure and the first dose of PEP. Studies in non-occupational populations with post-sexual exposure included patients who received PEP in a hospital in the U.K.^78^ or adults and children who requested PEP in the Netherlands^80^, individuals 15 years and older during a PEP consultation at three AIDS information centers in France;^79^ and adults who previously utilized PEP services due to sexual and other exposures via an internet medical platform in China and subsequently completed an online questionnaire.^75^

The evidence from these five studies suggests no difference in seroconversion among individuals who initiated PEP before or after 72 hours post-HIV exposure. The three cohort studies^78-80^ and the one cross-sectional study^75^ reported no events or a low number of events, limiting the ability to conduct statistical analyses, and those events are subject to confounding by additional high-risk exposures. One study^77^ reported a small sample size and another^75^ relied on self-reported data for PEP regimen and subsequent seroconversion.

# **F. KQ4 PEP Indications**

## **F.1. KQ4 Recommendation and Justifications**

KQ 4 What is the risk of a patient with undetectable HIV viral load transmitting to healthcare personnel with an occupational percutaneous and/or mucous membrane exposure?

- Use shared decision-making involving the HCP following occupational exposure to a patient with an undetectable serum viral load when considering whether to forego initiation of PEP or discontinue PEP early. **Recommendation,** Moderate Confidence

**Table S8.** Recommendation Jjustification^3^ for IM-administered cabotegravir-based ART regimens

| **Component** | **Justification** |
| --- | --- |
| Supporting evidence | Seven cohort studies^81-87^ one case series,^88^ and one case report^89^ |
| Level of confidence in evidence | Moderate: Despite concerns over small sample sizes and unclear compliance with PEP, it is not anticipated that these findings will change. |
| Benefits | The literature suggested no difference in infections among those who did and did not initiate PEP after an exposure to a source patient with an undetectable viral load. The literature indicated that there are no transmissions from an HIV positive partner with an undetectable viral load to their long-term HIV negative partner. An important benefit to the use of PEP is that it may reassure HCP who have sustained an occupational exposure despite the undetectable serum viral load in the source patient. |
| Risks and harms | Harms were not retrieved from the literature. |
| Resource use | No retrieved evidence on resource use, however, the reduction in PEP use will conserve resources. |
| Benefit-Harm Assessment | The benefits and harms are specific to the needs of the individual which indicates shared decision-making is an optimal approach. |
| Value Judgements | Values include reducing HCP anxiety and improving the mental and physical health of the HCP and aligning recommendations for ease of implementation. |
| Intentional Vagueness | The verb *offer* is chosen because the final decision of initiating PEP is a decision shared by the occupational health clinic and the HCP. |
| Exceptions | None |

## **F.2. KQ4 Summary of Findings and Evidence Evaluation**

No evidence was retrieved by the literature search that could be summarized or evaluated.

## **F.3. KQ4 Narrative Summary of Evidence**

The literature search retrieved one cohort study,^82^ one case series,^88^ and one case report^89^ examining the risk of a patient with undetectable HIV viral load transmitting to healthcare personnel with an occupational percutaneous and/or mucous membrane exposure. Occupational populations included healthcare personnel with needlestick injuries at a German tertiary care hospital,^82^ dentists at a university hospital in Japan,^88^ and a female healthcare worker with a needlestick injury from the U.S.^89^ The literature search retrieved six additional cohort studies^81,83-86^ reporting the outcome of HIV seroconversion in non-occupational populations. Non-occupational populations included children with community-associated needlestick injuries or exposures to other potentially contaminated objects in Canada,^83^ patients of all ages with blood or sexual exposures in Denmark,^84^ and sexual exposures among adult heterosexual partners^85,86^ from various European countries.

Evidence from the nine included studies^81-89^ suggest there is no risk of a patient or person with undetectable HIV viral load transmitting to others via occupational or non-occupational exposures. Importantly, the three cohort studies,^81-83^ one case series,^88^ and one case report^89^ that include needlestick, blood, and occupational exposures reported no seroconversions among 767 participants. However, all five^81-83,88,89^ were subject to confounding by PEP use and there were no events reported across these studies. Four studies^84-87^ that include sexual exposures reported no^87^ or a low number of seroconversions,^84,86^ or no within-couple phylogenetically linked seroconversions.^85,86^ Three of these studies^84-86^ are subject to confounding due to repeated high-risk exposures among those who seroconverted and one^84^ due to PEP use. Three studies reporting on non-occupational exposures^83,84,87^ are subject to sampling bias due to loss to follow-up.

An additional technical report^90^ identified by subject matter experts in the grey literature reported on significant occupational exposures to HIV among healthcare personnel in the U.K.; however, the study did not report the viral load of source patients and therefore did not meet the inclusion criteria for this systematic review. The study reported only one confirmed seroconversion during a period of more than 20 years that included 8,292 occupational exposures among healthcare personnel where the source patient was either HIV-positive or their status was unknown. Of those with an occupational exposure, there were 3,385 who started PEP. Of note, occupationally acquired HIV is rare and there has only been no confirmed cases of transmission since 1999 among HCP providing patient-facing care.^61^

# **G. KQ5 Fourth Generation HIV Tests**

## **G.1. KQ5 Recommendation and Justifications**

KQ 5 What is the window of time until HIV antibodies can be detected using a HIV Ag/ab test, among healthcare personnel with an occupational exposure?

- Perform final HIV tests using lab-based HIV Ag/Ab combination immunoassay and qualitative nucleic acid test (NAT) at week 12 post-exposure of exposed HCP. **Recommendation,** Moderate Confidence

**Table S9.** Recommendation justification^3^ for fourth generation HIV tests

| **Component** | **Justification** |
| --- | --- |
| Supporting evidence | Two diagnostic accuracy studies^64,65^and two cohort studies^91,92^ |
| Level of confidence in evidence | Moderate: The evidence supporting the window of time for an HIV Ag/ab test to detect an infection is unlikely to change due to the decreasing number of patients who are not on PrEP or PEP who can be tested for HIV infection. The utilization of nucleic acid in conjunction with HIV Ag/ab testing is standard of care^43^ that is supported by one diagnostic accuracy study in this review^64^. |
| Benefits | The reduced window to detection^93^ offers a more rapid return of changes in HIV status. |
| Risks and harms | There is the possibility of a smoldering infection that goes undetected. Experts on the panel determined that these cases are increasingly rare in the current prophylactic and testing paradigm. |
| Resource use | There was no evidence retrieved on resource use. |
| Benefit-Harm Assessment | The benefits outweigh the harms. |
| Value Judgements | Values include providing a wider window of detection than the minimum in order to assure detection of HIV antibodies, harmonizing with global testing recommendations. Utilization of the more sensitive nucleic acid test in conjunction with the HIV Ag/ab test is standard of care. |
| Intentional Vagueness | The window of time estimated in this recommendation is not specified but includes estimated washout time for PEP of up to four weeks,^94-97^ the estimated time to seroconversion of three to four weeks,^93,98^ and the doubling of this duration to account for the uncertainty in the studies and to decrease the risk that a case is missed.^99^ |
| Exceptions | None |

## **G.2. KQ5 Summary of Findings and Evidence Evaluation**

**Table S10.** GRADE Table: Duration to positive test/ detection when using fourth generation HIV tests

| **Outcome** | **Summary** | **Studies** | **Confidence** |
| --- | --- | --- | --- |
| Duration to positive test/detection | Data suggests the duration to positive test is less than 3 months post HIV exposure when using fourth-generation HIV tests. | N = 222 specimen,^64^  N = 1,241 assays^65^  2 Laboratory Studies | Moderate^[[5]](#footnote-5)^ |
| HIV Seroconversions | Data from two cohort studies^31, 32^ of high-risk sexual exposures is insufficient to determine whether fourth generation tests will detect HIV seroconversion earlier than 3 months post HIV exposure when using fourth-generation HIV tests. | N = 714 non-occupational participants  2 Cohort studies^91,92^ | Low^[[6]](#footnote-6)^ |

## **G.3. KQ5 Narrative Summary of Evidence**

The original literature search strategy did not retrieve studies using fourth generation HIV Ag/ab tests to monitor for the emergence of an HIV infection after three months post occupational HIV exposure among healthcare personnel. The search retrieved two cohort studies^91,92^ reporting HIV seroconversion using fourth generation testing in non-occupational populations. These populations included people evaluated at an HIV infection care center in France after sexual exposures to HIV^91^ and patients seeking PEP at two STI clinics in Canada.^92^ Both studies reported rates of seroconversion ranging from 2.3%^91^ to 4.5%^92^ after 3 months post-HIV exposure. However, one study^91^ reported seroconversions occurring more than six months after nPEP and the other^92^ captured seroconversions within 12 months of follow-up. In one study,^92^ four of the five patients who seroconverted tested negative at four months after completing PEP, while the other patient did not return for follow-up testing. None of the five patients who seroconverted returned for follow-up testing at two or four months in the other study^91^, and three patients had presented multiple times for nPEP evaluation.

The updated literature search strategy retrieved two diagnostic accuracy studies^64,65^ examining the duration of time between HIV exposure or transmission to detection of infection. One study^64^ evaluated the time between an exposure and when fourth generation Ag/ab tests can accurately detect the presence of an HIV infection and found that the median duration to detection for antibody/antigen laboratory tests was 17.8 days (IQR: 13.0 – 23.6) and that 99% of HIV-infected persons would be detectable within 45 days of exposure, which was the lowest number of days among all included tests. The other diagnostic accuracy study^65^ evaluated the number of days from infection to first positive third- or fourth-generation test result and found that the median duration to detection of HIV infection was 18 days (IQR: 16-24) for fourth-generation tests and 22 days (IQR: 19 – 25) for third-generation tests. This study^65^ also found that the probability of a false-negative result is 0.01 at 42 days for fourth-generation tests (99% CI: 0.05 to 0.027) and 85 days post-exposure for third-generation tests (99% CI: 0.002 – 0.022). Importantly, this study was conducted using the Aptima HIV-1 RNA nucleic acid amplification test (NAT) to estimate the inter-test reactivity interval which was then combined with simulated data to estimate the eclipse period – or window of detection for the fourth generation HIV ag/ab test. This study reported that the median duration of the time between HIV acquisition or exposure and Aptima reactivity was 11.5 days, and the 99th percentile of the eclipse period between HIV acquisition and test reactivity was 33 days.

The evidence from two cohort studies is insufficient to determine the risk of breakthrough infection after 3 months post occupational HIV exposure when using a fourth generation HIV Ag/ab test. Importantly, the evidence from two diagnostic accuracy studies using commercially and publicly available assays suggests the window of time to detection of HIV antibodies is less than 3 months post HIV exposure when using a fourth-generation HIV test.^64,65^ The two cohort studies^91,92^ did not conduct statistical analyses and had a low number of events. One study reported high attrition^91^ and the other^92^ did not report the number of people who completed follow-up testing. Additionally, the accuracy of the duration to detection in both diagnostic accuracy studies^64,65^ depends on the accuracy of the estimates of the simulated eclipse periods. One of these studies^64^ assumed the included specimen used for analysis were infected with HIV-1 subtype B; other HIV subtypes may have different durations to detection. The other study^65^ used data from HIV tests conducted between 1981 and 2006, and fourth-generation tests have improved since then.

# **H. Management and Testing for HCP on PrEP**

## **H.1 Management of Occupational Exposures in HCP on PrEP: Recommendation and Justifications**

- Use shared decision-making involving the exposed HCP taking PrEP when deciding whether to forego initiation of PEP or discontinue PEP early. (Good Practice Statement, Indirect Evidence)

**Table S11**. Recommendation fustification^3^ for shared decision-making involving the exposed HCP taking PrEP

| **Component** | **Justification** |
| --- | --- |
| Supporting evidence | Using shared decision-making enables patients to weigh risks and benefits according to their personal values and preferences and is a standard of care in preventive care decisions.^70^ |
| Level of confidence in evidence | Not graded |
| Benefits | Benefits include enabling patients and providers to individualize decision-making in settings where risk of HIV infection via occupational exposure may be lessened by use of PrEP and decreasing the use of PEP regimen prescriptions. |
| Risks and harms | The risk for HIV seroconversion from not giving PEP to HCP adherent to PrEP has not been demonstrated but may theoretically be possible. |
| Resource use | In some situations, the decision may save the financial and material resources of using PEP in situations where it is either unlikely to be beneficial or not desired by the exposed HCP. |
| Benefit-Harm Assessment | The balance of benefits and harms is specific to exposed HCP, resulting in the need for shared decision making. |
| Value Judgements | Values include promoting decision-making autonomy of exposed HCP, reducing patient harm, reducing pill burden, reducing side effects, and prioritizing adherence. |
| Intentional Vagueness | None |
| Exceptions | None |

## **H.2 Repeat Testing for Exposed HCP on PrEP: Recommendation and Justifications**

- Perform an additional baseline qualitative nucleic acid test (NAT) **only** for exposed HCP who have received cabotegravir-based PrEP in the past 12 months. (Good Practice Statement, Indirect Evidence)

**Table S12**. Recommendation justification^3^ for additional baseline qualitative NAT test for exposed HCP who have received cabotegravir-based PrEP in the last 12 months.

| **Component** | **Justification** |
| --- | --- |
| Supporting evidence | PrEP regimens that contain long-acting cabotegravir have a prolonged elimination half-life. ^55^ |
| Level of confidence in evidence | Not graded |
| Benefits | Providing opportunity to diagnose HIV at baseline among HCP on long acting cabotegravir PrEP would enable initiation of HIV treatment and obviate the need for PEP. |
| Risks and harms | None |
| Resource use | There will be an increase in resource use associated with the additional cost of HIV NAT test. |
| Benefit-Harm Assessment | The benefits outweigh the harms by enabling detection of new HIV diagnosis without any risk to HCP on cabotegravir-based PrEP . |
| Value Judgements | Values include diagnosing HIV in this population and preventing unnecessary use of PEP for those diagnosed with HIV. |
| Intentional Vagueness | None |
| Exceptions | None |

**Table S13.** GRADE Table: HIV seroconversion following exposure to source patients with undetectable HIV viral load

| **Outcome** | **Summary** | **Studies** | **Confidence** |
| --- | --- | --- | --- |
| HIV Seroconversion | Evidence from one cohort, one case report, and one case series reporting on PEP administration and completion for occupational exposures with undetectable or unknown HIV status indicates no risk of transmission to HCP. This is further supported by six cohort studies in non-occupational populations where there are no transmissions from a partner with an undetectable viral load to their HIV-negative partner. | N = 525 occupational exposures  N = 1865 non-occupational participants  7 cohort studies^81-87^  1 case series^77^  1 case report^89^ | Moderate^[[7]](#footnote-7)^ |

# **I. Included Evidence**

## **I.1 Extracted Evidence from Individual Studies**

**Table S14**. Select extracted data from studies reporting antiretroviral therapy outcomes of interest

| **Study** | **Population and setting** | **Intervention** | **Definitions** | **Results** |
| --- | --- | --- | --- | --- |
| **Author**: Bernasconi^81^  **Year:** 2001  **Data extractor:** DOS  **Reviewer:** CNS  **KQ(s):** 4  **Study design:** Cohort  **Study objective:** To analyze the data from Swiss nationwide voluntary reporting on nPEP by prescribing physicians.  **Limitations:**  Confounding due to PEP use | **Population:** N = 176  **Setting:** Community  **Location:** Switzerland  **Study dates:** December 1997 – March 2000  **Matching:** None  **Inclusion criteria:** Persons who were prescribed HIV PEP for non-occupational exposures.  **Exclusion criteria:** NR | **Intervention group:** n = 19  Patients exposed to source patient with known HIV status and known viral load <400 copies/mL [19/40 (47%)]   - **ART regimen:** NR, however most exposed patients received zidovudine/lamivudine alone [8/167 (4.8%)] or in combination with nelfinavir [57/167 (34.1%)], indinavir [38/167 (22.8%)], or nevirapine [31/167 (18.6%)]. Others received other triple combinations [23/167 (13.8%)] or zidovudine with didanosine [1/167 (0.6%)]. - **Viral load of source patient:** <400 copies/mL   **Control group:** n = 21  Patients exposed to source patient with known HIV status and known viral load ≥400 copies/mL [21/40 (53%)]   - **ART regimen:** NR - **Viral load of source patient:** ≥400 copies/mL   **Exposure assignment or ascertainment:** Information was collected via standardized questionnaire completed by physicians on a voluntary basis | **Outcome definitions:**  Seroconversion: Positive HIV serology at 3, 6, or 9 months after exposure  **Case ascertainment:** Passive surveillance system required physicians to provide follow-up HIV serology at 3, 6, and 9 months after exposure  **Sampling methods:** NR  **Diagnostic tests:** Serology  **Comments:** This study considers <400 copies/mL as undetectable. | **HIV-related outcomes:**  Seroconversion: No seroconversions have been reported among this group of HIV-exposed persons.  **Other related outcomes:** NR  **Adverse events:** NR |
| **Author**: Delaney^64^  **Year:** 2017  **Data extractor:** DOS  **Reviewer:** CNS  **KQ(s):** 5  **Study design:**Basic science  **Study objective:** To develop window period estimates for categories of HIV tests.  **Limitations:** | **Population:**  N = 222 specimen  N = 20 HIV immunoassays  **Setting:** Laboratory  **Location:** U.S.  **Study dates:** NR  **Matching:** NA  **Inclusion criteria:** U.S. Food and Drug Administration-approved HIV immunoassays  **Exclusion criteria:** NR | **Intervention group:** n = 222 specimen   - **ART regimen:** NR - **HIV test used:** Antibody/antigen laboratory   **Control group:** n = 222 specimen   - **ART regimen:** NR - **HIV test used:** HIV-1 RNA nucleic acid test   **Exposure assignment or ascertainment:** NR | **Outcome definitions:**  Duration to detection: The time between an exposure to HIV and when an HIV test can accurately detect the presence of an infection  Inter-test reactivity interval: The time period between detection of HIV infection using HIV-1 RNA nucleic acid test and using other tests  **Case ascertainment:** Commercially available specimens were determined to be in the early stages of seroconversion after infection with HIV-1. Specimens were presumed to be infected with HIV-1 subtype B.  **Sampling methods:** Serial plasma specimens were collected from 25 donors from the U.S between 1994 and 2000.  **Diagnostic tests:** Antibody/antigen laboratory, IgG/IgM-sensitive laboratory, IgG-sensitive rapid screening, IgG-sensitive supplemental, and Western blot (viral lysate).  **Comments:** The accuracy of the window periods depends on the accuracy of the estimate of the simulated eclipse periods. | **HIV-related outcomes:**  Duration to detection, median days (IQR):   - Antibody/antigen laboratory: 17.8 (13.0-23.6) - IgG/IgM-sensitive laboratory: 23.1 (18.4-28.8) - IgG-sensitive rapid screening: 31.1 (26.2-37.0) - IgG-sensitive supplemental: 33.4 (28.5-39.2) - Western blot (viral lysate): 36.5 (31.0-43.2)     Duration to detection, 99^th^ percentile days:   - Antibody/antigen laboratory: 44.3 - IgG/IgM-sensitive laboratory: 49.5 - IgG-sensitive rapid screening: 56.7 - IgG-sensitive supplemental: 58.2 - Western blot (viral lysate): 64.8     Inter-test reactivity interval, median days (95% CI):   - Antibody/antigen laboratory: 5.9 (3.9-7.9) - IgG/IgM-sensitive laboratory: 11.9 (9.6-14.1) - IgG-sensitive rapid screening: 19.5 (16.2-22.8) - IgG-sensitive supplemental: 21.7 (17.4-26.1) - Western blot (viral lysate): 24.8 (18.1-31.4)   **Other related outcomes:** NR  **Adverse events:** NR |
| **Author**: Do^77^  **Year:** 2003  **Data extractor:** DOS  **Reviewer:** CNS  **KQ(s):** 3  **Study design:** Case series  **Study objective:** To summarize national surveillance data on HIV infection and AIDS among HCP, focusing on those with documented occupationally acquired HIV infection.  **Limitations:** no major | **Population:**  N = 57  **Setting:** Healthcare or laboratory  **Location:** U.S.  **Study dates:** 1985 – December 2001  **Matching:** None  **Inclusion criteria:** HIV-infected HCP with or without AIDS who were determined to have possible occupationally acquired infection.  **Exclusion criteria:** NR | **Intervention group:** n ≥ 1   - **ART regimen:** Zidovudine 1,200 mg for 3 weeks - **Interval between HIV exposure and PEP initiation:** 192 hours   **Control group:** n ≥ 7   - **ART regimen:** Zidovudine 600-1,000 mg ranging from 1 dose to 65 days; didanosine for 3 days and stavudine and nevirapine for 4 weeks - **Interval between HIV exposure and PEP initiation:** 30 minutes – 8 hours   **Exposure assignment or ascertainment:** Data from the HIV/AIDS Reporting System and the National Surveillance for Occupationally Acquired HIV Infection | **Outcome definitions:**  *Occupational HIV infection:* Documented evidence of seroconversion or genetically similar strains of viruses. Seroconversion was defined as a serum specimen negative for HIV antibodies up to 1 year before or 1 month after the occupational exposure, and a subsequent serum sample that was positive for HIV antibodies within 1 year of the exposure. This also included individuals infected with HIV strains shown to be related to the occupational source through DNA sequencing techniques.  **Case ascertainment:** Data from the HIV/AIDS Reporting System and the National Surveillance for Occupationally Acquired HIV Infection  **Sampling methods:** NR  **Diagnostic tests:** Serologic testing  **Comments:** None | **HIV-related outcomes:**  *Seroconversion:* Of eight HCP with occupationally acquired HIV infection who received PEP, one initiated prophylaxis 192 hours after exposure. The remaining seven initiated 30 minutes to 8 hours after exposure.  **Other related outcomes:** NR  **Adverse events:** NR |
| **Author**: Gantner^71^  **Year:** 2020  **Data extractor:** CNS    **Reviewer:** DOS  **KQ(s):** 1  **Study design:** Cohort    **Study objective:** To identify predictors of PEP completion, including PEP regimen, in the Dat’AIDS prevention cohort.  **Limitations:** no major | **Population:**  N = 1,570  **Setting:** 23 HIV infection care centers involved in PEP prescription  **Location:** France  **Study dates:** 2004 – 2017  **Matching:** None  **Inclusion criteria:** HIV-noninfected individuals evaluated for PEP after sexual risk behavior from the Dat’AIDS prevention cohort and gave written consent.  **Exclusion criteria:** NR | **Intervention group:** n = 704   - **ART regimen:** TDF/FTC+DTG     **Control group:** n = 866   - **ART regimen:** TDF/FTC+RAL     **Exposure assignment or ascertainment:** PEP regimen was determined by electronic medical records | **Outcome definitions:**  Early discontinuation: PEP discontinuation <20 days after initiation of PEP regimen (reported discontinuations and losses to follow-up)  **Case ascertainment:** HIV test performed at baseline and weeks 8 and 16 for individuals receiving PEP  **Sampling methods:** NR  **Diagnostic tests:** NR  **Comments:** None | **HIV-related outcomes:**  Seroconversion:   - Intervention: 0/704 - Control: 0/866     **Other related outcomes:**  Early discontinuation:   - Intervention: 87.2% - Control: 96.4%     **Adverse events:** NR |
| **Author**: Gantner^91^  **Year:** 2015  **Data extractor:** CNS  **Reviewer:** DOS  **KQ(s):** 5  **Study design:** Retrospective cohort  **Study objective:** To identify predictors of HIV-testing follow-up completion among patients demanding nPEP, to determine trends in the sexual exposures/condom use, and to describe the nPEP experience.  **Limitations:**   - Confounding (PEP use, compliance) - Sampling bias (attrition, missing follow-up information) | **Population:**  N = 602 patients with 646 sexual exposures to HIV  **Setting:** University hospital and HIV-infection care center  **Location:** France  **Study dates:** January 2009 – December 2013  **Matching:** NA  **Inclusion criteria:** Patients evaluated after sexual exposure to HIV.  **Exclusion criteria:** NR | **Intervention group:** n = 602 patients with 646 sexual exposures   - **ART regimen:** 507/646 (78%) Lamivudine/zidovudine (150/300mg twice a day) for 28 days plus nevirapine for the first 4 days only (200mg once daily) - **HIV test used:** 4^th^ generation ELISA combined antigen-antibody laboratory assay   **Control group:** n = NA   - **ART regimen:** NA - **HIV test used:** NA   **Exposure assignment or ascertainment:** Electronic medical files | **Outcome definitions:**  *Seroconversion:* HIV positive test result at week 8 or 16 for patients taking nPEP or week 6 for patients not taking nPEP  *Follow-up completion:* HIV testing performed at baseline and weeks 8 and 16 for patients taking nPEP and baseline and week 6 for patients not taking nPEP  **Case ascertainment:** HIV-testing performed at baseline and weeks8 and 16 for patients taking nPEP or baseline and week 6 for patients not taking nPEP  **Sampling methods:** NR  **Diagnostic tests:** 4^th^ generation ELISA combined antigen-antibody laboratory assay  **Comments:** None | **HIV-related outcomes:**  *Seroconversion:* No documented nPEP failure was identified. However, 5/217 (2.3%) MSM nPEP recipients for unprotected anal receptive intercourse subsequently seroconverted to HIV more than 6 months after nPEP. None of the five MSM achieved follow-up completion of HIV testing at weeks 8 and 16. Three of the five MSM presented more than once for nPEP evaluation, indicating ongoing risk behaviors.  **Other related outcomes:**  *Follow-up completion:* HIV-testing follow up completion rate was 30%.  **Adverse events:** NR |
| **Author**: Grzeszczuk^87^  **Year:** 2017  **Data extractor:** CNS  **Reviewer:** DOS  **KQ(s):** 4  **Study design:** Cohort  **Study objective:** To analyze epidemiological characteristics of HIV serodiscordant couples and to observe current attitudes towards safe sex, procreation, and efficacy of treatment.  **Limitations:** no major | **Population:**  N = 52 couples  **Setting:** HIV out-patient clinic of a hospital for infectious diseases  **Location:** Poland  **Study dates:** 2014 — NR (1 year period)  **Matching:** None  **Inclusion criteria:** Consecutive HIV positive persons attending the clinic in 2014 and their HIV negative partners  **Exclusion criteria:** NR | **Intervention group:** n = 42 HIV-negative patients with HIV-positive partners with viral load <50 copies/ml   - **ART regimen:** NR - **Viral load of source patient:** HIV RNA <50 copies/ml   **Control group:** n = 10 HIV-negative patients with HIV-positive partners with viral load >50 copies/ml   - **ART regimen:** NR - **Viral load of source patient:** HIV RNA >50 copies/ml   **Exposure assignment or ascertainment:** Data obtained from medical files | **Outcome definitions:**  *Seroconversion:* HIV transmission  **Case ascertainment:** Medical files  **Sampling methods:** NR  **Diagnostic tests:** NR  **Comments:** None | **HIV-related outcomes:**  *Seroconversion:* During one year of observation, 18 (35%) patients had detectable HIV RNA, which ranged from 61 to 182 292 copies/ml (median: 15 037 copies/ml). No seroconversions were reported in medical files, but only half of HIV negative partners were tested regularly.  **Other related outcomes:** NR  **Adverse events:** NR |
| **Author**: Himmelreich^82^  **Year:** 2013  **Data extractor:** CNS  **Reviewer:** DOS  **KQ(s):** 4  **Study design:** Cohort  **Study objective:** To evaluate needlestick injuries (NSIs) reported to accident insurance doctors and to establish their frequency, in order to obtain an overview of the infection-related and epidemiological factors in the disease burden resulting from NSIs.  **Limitations:**  Confounding due to PEP use | **Population:** N = 519    51/449 (11.4%) index patients were HIV-positive  **Setting:** Tertiary-care university hospital  **Location:** Germany  **Study dates:** Mid-October 2010 – mid-April 2012  **Matching:** None  **Inclusion criteria:** HCP with a NSI, defined as puncture wounds, cuts, or scratches inflicted by medical instruments intended for cutting or puncturing (cannulae, lancets, scalpels, etc.) that may be contaminated with a patient’s blood/other bodily fluids or contact of blood with nonintact skin and contact with mucous membranes, reported to accident insurance doctors.  **Exclusion criteria:** NR | **Intervention group:** n ≥ 16  HCP exposed to HIV-positive source patients with an undetectable viral load of ≤20 copies/mL   - **ART regimen:** NR - **Viral load of source patient:** 16/19 (84.2%) had ≤20 copies/mL   **Control group:** n ≥ 3  HCP exposed to HIV-positive source patients with a viral load of >20 copies/mL   - **ART regimen:** NR - **Viral load of source patient:** 3/19 (15.8%) had >20 copies/mL (up to 7,360,000 copies/mL)   **Exposure assignment or ascertainment:** Based on accident insurance doctors’ reports and occupational follow-up examinations | **Outcome definitions:**  Seroconversion: Testing positive for HIV on day 14 or after day 28  **Case ascertainment:** HCP receiving PEP were followed up on day 14 and after day 28  **Sampling methods:** Blood sample  **Diagnostic tests:** Serological anti-HIV testing  **Comments:** HIV regimens included a daily dose of tenofovir 300mg/emtricitabine 200mg and lopinavir 800mg/ritonavir 200mg for 28 days. Another antiviral therapy was administered to those where the source patient had HIV resistance or if the drug combination was intolerable. | **HIV-related outcomes:**  Seroconversion: No transmission of infection occurred during the follow-up period.  **Other related outcomes:** NR  **Adverse events:** NR |
| **Author**: Kordy^83^  **Year:** 2017  **Data extractor:** CNS  **Reviewer:** DOS  **KQ(s):** 4  **Study design:** Cohort  **Study objective:** To retrospectively analyze the epidemiology, management and outcome of children following community exposures associated with potential risk of blood-borne pathogen transmission in the Greater Toronto Area.  **Limitations:**   - Confounding due to PEP use   Selection bias due to loss to follow-up | **Population:** N = 66  **Setting:** Community  **Location:** Canada  **Study dates:** January 2001 – December 2014  **Matching:** None  **Inclusion criteria:** Children <19 years old with a community-associated needlestick injury or exposures to other objects potentially contaminated with body fluids that can contain blood-borne pathogens.  **Exclusion criteria:** Sexual and hospital inpatient exposures. | **Intervention group:** n ≥ 4  Children with known source whose viral load <50 copies/mL   - **ART regimen:** 2 were on 4 weeks of zidovudine, lamivudine, and lopinavir/r; 1 declined treatment; and 1 discontinued PEP within 24 hours after risk assessment - **Viral load of source patients:** <50 copies/mL   **Control group:** n ≥ 2  Children with known source whose viral load >50 copies/mL   - **ART regimen:** 1 was on 6 weeks of zidovudine, lamivudine, and 4 weeks of nevirapine; and 1 was not given ART - **Viral load of source patients:** >50 copies/mL (up to 1600 copies/mL)   **Exposure assignment or ascertainment:** Patients were identified by reviewing all medical records of those who had HIV tests submitted through the microbiology laboratory. | **Outcome definitions:**  Seroconversion: HIV positive at 6 weeks, 3 months, or 6 months follow-up  **Case ascertainment:** HIV test results were ascertained through the microbiology laboratory  **Sampling methods:** NR  **Diagnostic tests:** Serology test  **Comments:** None | **HIV-related outcomes:**  Seroconversion: None of the children who completed follow-up seroconverted. One child exposed to source patient with viral load <50 was lost to follow-up and HIV status is unknown.  **Other related outcomes:**  Adherence:   - Intervention: 2/2 (100%) - Control: 1/1 (100%)   **Adverse events:** NR |
| **Author**: Kumar^72^  **Year:** 2017  **Data extractor:** CNS  **Reviewer:** DOS  **KQ(s):** 1  **Study design:** Cohort study  **Study objective:** To compare two, three, and four-drug regimens, and their associated patient outcomes at the clinic.  **Limitations:**   - Confounding (compliance with PEP) | **Population:**  N = 19  **Setting:** Hospital  **Location:** Canada  **Study dates:** January 1, 2013 – December 31, 2015  **Matching:** None  **Inclusion criteria:** All consecutive patients seen by the Sexual Assault and Partner Abuse Care Program nurses who accepted a PEP drug regimen.  **Exclusion criteria:** Patients with a history of IV drug use, men who have sex with men, immunocompromised patients, patients who are already HIV positive or already taking HIV PEP at the time of presentation, patients who were seen for isolated physical assault without a sexual component, patients whose initial assessment for PEP was done at another institution, and patients seen in 2014. | **Intervention group:** n = 6  Participants received a four-drug PEP regimen at their initial presentation and were given a 28-day intervention regimen upon their first follow-up visit (3-7 days later). Follow up was scheduled frequently thereafter to dispense HIV PEP in 1-2 week quantities.   - **ART regimen:** Emtricitabine/Tenofovir +Dolutegravir     **Control group:** n = 13  Participants received a four-drug PEP regimen at their initial presentation and were given a 28-day control regimen upon their first follow-up visit (3-7 days later). Follow up was scheduled frequently thereafter to dispense HIV PEP in 1-2 week quantities.     - **ART regimen:** Emtricitabine/Tenofovir +Raltegravir     **Exposure assignment or ascertainment:** Medical records | **Outcome definitions:**  *Seroconversion:* HIV positive test at follow-up testing  **Case ascertainment:** HIV serology testing at initial visit, 3 weeks, and 3 months. Follow up data was obtained from nursing notes and documentation, as well as medical records.  **Sampling methods:** NR  **Diagnostic tests:** HIV serology testing  **Comments:** None | **HIV-related outcomes:**  *Seroconversion among those accepting PEP at 3 months and later:*   - Intervention: 0/6 - Control: 0/13     **Other related outcomes:** NR    **Adverse events:** NR |
| **Author**: Lunding^84^  **Year:** 2010  **Data extractor:** CNS  **Reviewer:** DOS  **KQ(s):** 4  **Study design:** Cohort  **Study objective:** To describe the incidence of the indications for, and the compliance with, PEP use after sexual exposure to HIV in Denmark from the introduction in 1998 up till 2006.  **Limitations**:   - Sampling bias (attrition) | **Population:** N = 374  **Setting:** 8 infectious disease clinics that treat HIV patients  **Location:** Denmark  **Study dates:** 1998 – 2006  **Matching:** None  **Inclusion criteria:** All cases of PEP after blood or sexual exposure to HIV.  **Exclusion criteria:** NR | **Intervention group:** n ≥ 51  Patients exposed to source patient known to be HIV-positive with viral load <50 copies/mL   - **ART regimen:** 3 drug regimens of 2 NRTI’s and 1 PI/boosted PI for 4 weeks, which included zidovudine + lamivudine + lopinavir/ritonavir, zidovudine + lamivudine + indinavir, zidovudine + lamivudine + efavirenz, zidovudine + lamivudine + nelfinavir, or other regimens. Treatments were individualized in case of knowledge of resistance in the source patient of adverse events. - **Viral load of source patient:** 51/221 (23.1%) had <50 copies/mL; data was missing for 124/221 (56.1%)   **Control group:** n ≥ 46  Patients exposed to source patient known to be HIV-positive with viral load >50 copies/mL   - **ART regimen:** 3 drug regimens of 2 NRTI’s and 1 PI/boosted PI for 4 weeks, which included zidovudine + lamivudine + lopinavir/ritonavir, zidovudine + lamivudine + indinavir, zidovudine + lamivudine + efavirenz, zidovudine + lamivudine + nelfinavir, or other regimens. Treatments were individualized in case of knowledge of resistance in the source patient of adverse events. - **Viral load of source patient:** 46/221 (20.8%) had >50 copies/mL; data was missing for 124/221 (56.1%)   **Exposure assignment or ascertainment:** Danish PEP registry data from structured questionnaires or medical records | **Outcome definitions:**  Seroconversion: HIV positive at 3- and 6-months follow-up  **Case ascertainment:** Danish PEP registry data  **Sampling methods:** NR  **Diagnostic tests:** NR  **Comments:** None | **HIV-related outcomes:**  Seroconversion:   - at 3 months: 1/374 (0.3%)   - seroconversion attributed by authors to repeated exposures during and after completion of PEP. - at 6 months: 0/374     **Other related outcomes:**  Complied with HIV testing:   - after 3 months: 191/374 (51%) - after 6 months: 75/374 (20%)   Adherence:  Completed: 244/374 (65%)  Interrupted” 35/374 (9%)  Missing data: 95/375 (25%).  **Adverse events:** NR |
| **Author**: Mayer^74^  **Year:** 2022  **Data extractor:** CNS    **Reviewer:** DOS  **KQ(s):** 1  **Study design:** Cohort study  **Study objective:** Open-label phase IV study to test the safety, tolerability, and acceptability of the fixed dose combination of Bictegravir, emtricitabine, and tenofovir alafenamide (BIC/FTC/TAF) as PEP for individuals who may have been exposed to HIV when they engaged in condomless intercourse.  **Limitations:** no major | **Population:**  N = 152    **Setting:** Community health center  **Location:** Massachusetts, U.S.  **Study dates:**  Intervention group: August 2018 – March 2020    Control group: March 2008 – March 2010  **Matching:** None  **Inclusion criteria:** Intervention group: Participants who believed they were recently exposed to HIV were recruited through referrals from primary care providers and a hot line in a community health center specializing in HIV and PEP care, and via self-referral after an online and in-person community education campaign. Eligible participants needed  to present for PEP within 72 hours of a high-risk exposure, consent to the trial, and consent to prospective monitoring over a three-month period.  Control group: Participants recruited in a previously conducted phase IV study from March 2008-March 2010.  **Exclusion criteria:** Individuals found to have acute HIV by a trained clinician or prior HIV infection using an HIV rapid test. | **Intervention group:** n = 52  August 2018 – March 2020   - **ART regimen:** BIC/FTC/TAF taken as one pill daily for 28 days     **Control group:** n = 100  Earlier phase IV study, March 2008 – March 2010   - **ART regimen:** Raltegravir plus emtricitabine/tenofovir (TDF/FTC/RAL)     **Exposure assignment or ascertainment:** Those in the intervention group were recruited for this phase IV study. Those in the control group were recruited for a previously conducted phase IV study. | **Outcome definitions:**  Seroconversion: A positive HIV antibody test at 4 weeks or 3 months post-initiation of a PEP regimen    Completed regimen as prescribed: Completed regimen without modification as determined by pill count and self-report    Stopped or modified regimen: NR    Lost to follow-up: NR    Adverse events: Participants were asked to contact site staff in the event they experienced any adverse events of concern and/or those that would lead them to discontinue the medication. Side effects and regimen acceptability information were self-reported using a standardized interview guide.  **Case ascertainment:** Testing was completed at baseline, 4 weeks, and 3 months.  **Sampling methods:** NR  **Diagnostic tests:** HIV rapid test, fourth generation antigen-antibody assay  **Comments:** None | **HIV-related outcomes:**  Seroconversion: None of the participants tested at four weeks and interviewed at three months became HIV-positive    **Other related outcomes:**  Adherence: Grade 2 fatigue was associated with early drug regimen discontinuation    Completed regimen as prescribed:   - Intervention: 90.4% - Control: 57.0% - p < 0.01     Stopped or modified regimen:   - Intervention: 0.0% - Control: 28.0% - p < 0.001     Lost to follow-up:   - Intervention: 9.6% - Control: 15.0% - p > 0.05     **Adverse events:**  Diarrhea/loose stool:   - Intervention: 7.7% - Control: 21.0% - p < 0.05     Fatigue:   - Intervention: 9.6% - Control: 14% - p > 0.05     Nausea/vomiting:   - Intervention: 15.4% - Control: 27.0% - p > 0.05     Headache:   - Intervention: 1.9% - Control: 15.0% - p < 0.01     Dizziness/lightheadedness:   - Intervention: 0.0% - Control: 10.0% - p < 0.01     Myalgia/arthralgia:   - Intervention: 1.9% - Control: 8.0% - p > 0.05     Creatinine clearance decreases were detected in 7 (13.5%) of the samples, with 4 of them being grade 2. Grade 1 elevated transaminases were seen in 2 (3.8%) participants. Each of these laboratory abnormalities were asymptomatic and resolved after the PEP regimen was completed on day 28.  Other adverse events that were reported by  one participant each that could have been related to the drug regimen included: headache, myalgias/arthralgias, rectal irritation, flatulence, forearm rash, dry mouth, nightmares, dysuria, and gastroenteritis. |
| **Author**: McCarty^78^  **Year:** 2011  **Data extractor:** DOS  **Reviewer:** CNS  **KQ(s):** 3  **Study design:** Cohort  **Study objective:** To assess the seven-year experience of PEP following sexual exposure in a regional center.  **Limitations:** high attrition | **Population:** N = 72  **Setting:** Hospital  **Location:** UK  **Study dates:** January 2003 – August 2009  **Matching:** None  **Inclusion criteria:** All patient who received PEP following sexual exposure from genitourinary medicine and accident & emergency departments.    **Exclusion criteria:** Patients who were offered PEP following sexual exposure and declined. | **Intervention group:** n = NR (8%)   - **ART regimen:** NR - **Interval between HIV exposure and PEP initiation:** ≥72 hours   **Control group:** n = NR (92%)   - **ART regimen:** NR - **Interval between HIV exposure and PEP initiation:** <72 hours   **Exposure assignment or ascertainment:** Clinic documentation and pharmacy records | **Outcome definitions:**  *Seroconversion:* Positive serology for HIV at three or six months  **Case ascertainment:** Patients attended for repeat serology at three and six months; patients attended subsequent genitourinary medicine appointments  **Sampling methods:** NR  **Diagnostic tests:** Serology  **Comments:** None | **HIV-related outcomes:**  *Seroconversion:* No seroconversions took place among patients, however only 25/72 (35%) patients attended for repeat serology at three months and only 18/72 (25%) attended at six months. A further eight patients tested negative for HIV at subsequent genitourinary medicine attendances.  **Other related outcomes:**  Only 50/72 (69%) patients attended follow-up after commencing PEP treatment.  *PEP completion:* 46/50 (92%)  All four patients who did not complete treatment were due to re-evaluation of risk and not a result of poor tolerability.  **Adverse events:** NR |
| **Author**: Nerad^89^  **Year:** 2001  **Data extractor:** CNS  **Reviewer:** DOS  **KQ(s):** 4  **Study design:** Case report  **Study objective:** To report a case of hypercholesterolemia that occurred 2 weeks after the start of highly active antiretroviral therapy (HAART) following a needlestick exposure to HIV in an HIV-negative health care worker who was receiving thyroid replacement therapy.  **Limitations:** no major | **Population:** N = 1  **Setting:** NR  **Location:** Illinois, U.S.  **Study dates:** NR  **Matching:** NA  **Inclusion criteria:** A 44-year-old female HCP who sustained a needlestick to her left forefinger from an HIV positive patient.  **Exclusion criteria:** NR | **Intervention group:** n = 1  The source patient had a CD4 count of 176 cells/ mm^3^ (16%), a rapid plasma reagin titer of 1:8, and presence of antibody to hepatitis B virus (surface IgG, core IgG) and hepatitis C virus.   - **ART regimen:** A four-week course of zidovudine (300 mg orally q12h) and lamivudine (150 mg orally q12h), indinavir (800 mg orally q8h). The indinavir was replaced after two weeks by nelfinavir (1250 mg orally q12h). - **Viral load of source patient:** 176 RNA copies/mm3   **Control group:** n = NA   - **ART regimen:** NA - **Viral load of source patient:** NA   **Exposure assignment or ascertainment:** Laboratory test results for virus load | **Outcome definitions:**  Seroconversion: HIV positive test at one year follow-up  **Case ascertainment:** HCP was tested at one year follow-up  **Sampling methods:** NR  **Diagnostic tests:** NR  **Comments:** None | **HIV-related outcomes:**  Seroconversion: At 1 year of follow-up, results of tests for rapid plasma reagin titer and HIV and hepatitis C infection remain negative.    **Other related outcomes:**  Adherence: HCP completed the 28-day regimen  **Adverse events:** NR |
| **Author**: O’Byrne^92^  **Year:** 2018  **Data extractor:** CNS  **Reviewer:** DOS  **KQ(s):** 5  **Study design:** Cohort  **Study objective:** To highlight the characteristics of patients diagnosed with HIV and determine what the data about these characteristics suggested when compared to guidelines about PEP, preexposure prophylaxis (PrEP), and HIV testing.  **Limitations:** no major | **Population:** N = 112  **Setting:** Two STI clinics  **Location:** Canada  **Study dates:** September 5, 2013 – September 4, 2016  **Matching:** NA  **Inclusion criteria:** All patients who sought PEP  **Exclusion criteria:** NR | **Intervention group:** n = 112  If a patient presented for PEP or was identified as eligible for PEP by clinic staff, the PEP nurse was paged and assumed care. This nurse ensured that the potential exposure occurred within the preceding 72 hours, was of significant risk (by risk of transmission and possibility of sero-discordance), and that the patient was likely uninfected with HIV.   - **ART regimen:** 72/112 (64.3%) One daily fixed-dose single tablet of emtricitabine 200mg/tenofovir DF 300mg plus raltegravir 400mg twice daily for 3-6 days with a possible PEP prescription to complete a 28-day course - **HIV test used:** Fourth-generation antigen/antibody combo assay   **Control group:** n = NA   - **ART regimen:** NA - **HIV test used:** NA   **Exposure assignment or ascertainment:** Data from patient files | **Outcome definitions:**  *Seroconversion:* HIV positive test result by fourth-generation assay after baseline  *Adherence:* Self-reported PEP completion  **Case ascertainment:** HIV testing was recommended at 6 weeks and 4 months after competing PEP. Follow-up test results were available in patient medical files. For patients who were ineligible for PEP, HIV testing and HIV status were checked using patient medical files and the Integrated Public Health Information System database for reportable STIs, but patients outside Ontario or using an anonymous test code could not be verified in the database. Anonymous HIV test results, negative test results, and positive results for participants who emigrated from Ontario were not captured.  **Sampling methods:** Blood  **Diagnostic tests:** Fourth-generation antigen/antibody combo assay  **Comments:** None | **HIV-related outcomes:**  *Seroconversion:* Five HIV seroconversions occurred within 12 months of PEP use. Four of these patients had a negative HIV test result at routine follow-up four months after completing PEP. One patient with no documented follow-up testing was diagnosed during routine testing about one year after taking PEP.  No patient who requested PEP but did not meet clinical criteria to start PEP was later diagnosed with HIV  **Other related outcomes:**  *Adherence:* The five participants that seroconverted within 12 months of PEP use self-reported that they completed the prescribed 28 days of PEP.  **Adverse events:** NR |
| **Author**: Rey^79^  **Year:** 2008  **Data extractor:** DOS  **Reviewer:** CNS  **KQ(s):** 3  **Study design:** Cohort  **Study objective:** To characterize physicians’ adherence to guidelines and adherence to nPEP prescription in individuals sexually exposed to HIV.  **Limitations:** no major | **Population:**  N = 884 patients  N = 910 sexual exposures  **Setting:** Three AIDS information centers  **Location:** France  **Study dates:** January 1, 2001 – December 31, 2002  **Matching:** None  **Inclusion criteria:** All individuals aged 15 years or more who reported a sexual exposure during a consultation for nPEP.  **Exclusion criteria:** NR | **Intervention group:** n = NR   - **ART regimen:** Four-week combination antiretroviral therapy (cART) or double combination therapy - **Interval between HIV exposure and PEP initiation:** >72 hours   **Control group:** n = NR   - **ART regimen:** Four-week cART or double combination therapy - **Interval between HIV exposure and PEP initiation:** <72 hours   **Exposure assignment or ascertainment:** Retrospective data collection of information at the first visit after exposure | **Outcome definitions:**  *Seroconversion:* Positive HIV test  **Case ascertainment:** Individuals were invited to come back at least once between 2^nd^ and 4^th^ subsequent weeks for biological assessment  **Sampling methods:** NR  **Diagnostic tests:** NR  **Comments:** None | **HIV-related outcomes:**  *Seroconversion:* Only one case, a 40-year-old woman, tested positive after completing nPEP. The patient was consulted more than 72 hours after a high-risk exposure.  **Other related outcomes:**  *Treatment completion:* 355/437 (81.2%)  Of the 82 individuals that discontinued PEP, 11 did so because of clinical side effects and two because of biological side effects (elevated transaminases and neutropenia).  **Adverse events:** NR |
| **Author**: Rodger^85^  **Year:** 2019  **Data extractor:** CNS  **Reviewer:** DOS  **KQ(s):** 4  **Study design:** Cohort  **Study objective:** To produce a similar level of evidence for transmission risk through condomless anal sex between men with suppressive ART (defined as HIV-1 RNA viral load <200 copies per mL) to that generated for heterosexual couples in PARTNER1.  **Limitations:** no major | **Population:**  N = 782 couples  **Setting:** 75 clinical sites  **Location:** 14 European countries  **Study dates:** September 15, 2010 – April 30, 2018  **Matching:** NA  **Inclusion criteria:** Gay male serodifferent couples (HIV-positive male on ART and their HIV-negative male partner) who were aged 18 years or older and reported having penetrative sex with each other without condoms in the month before enrollment and during study and who reported that they expected to have sex together again in the coming months. The HIV-positive partner must have reported that they expected to remain on ART, the HIV-negative partner was not on PEP or PrEP, and the HIV-1 RNA viral load in the HIV-positive partner was < 200 copies per mL for the past 12 months.  **Exclusion criteria:** HIV-negative partner (or the HIV-positive partner if the HIV-negative partner did not reply) reported no condomless sex, most recent viral load of HIV-positive partner >200 copies per mL, data on sexual behavior missing, no viral load available in the past year for each day in the time period, or no HIV test from the HIV-negative partner at the end of the time period or later in time. | **Intervention group:** n = 782  HIV-negative males who have sex with HIV-positive male partners with an undetectable viral load of <200 copies/mL   - **ART regimen:** None - **Viral load of source patient:** <200 copies/mL   **Control group:** n = NA   - **ART regimen:** NA - **Viral load of source patient:** NA   **Exposure assignment or ascertainment:** Current and recent plasma HIV-1 RNA load were recorded at baseline and at each visit every 6-12 months | **Outcome definitions:**  Seroconversion: HIV positive antigen-antibody test when tested every 6-12 months    Adherence: Self-reported adherence to ART  **Case ascertainment:** The HIV-negative partner was tested for HIV every 6-12 months. If positive, sequence testing was completed to determine if transmission is phylogenetically linked.  **Sampling methods:** Plasma and blood  **Diagnostic tests:** Combined HIV antigen-antibody test with subsequent sequence testing if positive  **Comments:** None | **HIV-related outcomes:**  Seroconversion:  Couples reported having condomless anal sex approximately 76,088 times during eligible couple-years of follow-up. There were 15/782 (1.9%) initially HIV-negative partners who became HIV-1 positive during eligible follow-up, but there were no within-couple phylogenetically linked transmissions. Thirteen individuals provided information about their presumed source of HIV infection and 10/13 (77%) reported recent condomless sex with men other than their study partner.    During noneligible couple-years of follow-up with viral load >200 copies per mL, couples reported having condomless sex a total of 810 times with zero phylogenetically linked transmissions.  **Other related outcomes:**  Adherence: For 1461/1593 (92%) eligible couple-years of follow-up, adherence was >90% according to the HIV-positive partner. It was not reported for 96/1593 (6%) couple-years of follow-up.  **Adverse events:** NR |
| **Author**: Rodger^86^  **Year:** 2016  **Data extractor:** DOS  **Reviewer:** CNS  **KQ(s):** 4  **Study design:** Cohort  **Study objective:** To follow serodifferent partnerships that have penetrative sex without condoms in which the HIV-positive partner is taking ART with a plasma HIV-1 RNA load <200 copies/mL and to study risk of HIV transmission through anal and vaginal sex in the absence of condom use.  **Limitations** no major | **Population:**  N = 888 couples  **Setting:** 75 clinical sites  **Location:** 14 European countries  **Study dates:** September 2010 – May 31, 2014  **Matching:** None  **Inclusion criteria:** HIV-positive people older than 18 years who were taking ART and their HIV-negative partner. The partners reported penetrative sex without using condoms together in the month before enrollment (during which period the HIV-negative partner was aware of the HIV status of the HIV-positive partner) and during the study period, and the partners expected to have sex together again in the coming months. The HIV-positive partner was expected to continue taking ART and the latest plasma HIV-1 RNA load was <200 copies/mL and not dated older than 12 month.  **Exclusion criteria:** HIV-negative partner did not have HIV test, reported use of PrEP or PEP or did not report condomless sex (or none reported by HIV-positive partner if the HIV-negative partner did not reply). The latest plasma HIV-1 RNA load in the positive partner was ≥200 copies/mL or was not available. | **Intervention group:** n = 548 heterosexual and n = 340 MSM participants  HIV-negative people who have sex with HIV-positive partners with an undetectable viral load of <200 copies/mL   - **ART regimen:** None - **Viral load of source patient:** <200 copies/mL   **Control group:** n = NA   - **ART regimen:** NA - **Viral load of source patient:** NA   **Exposure assignment or ascertainment:** Data on plasma HIV-1 RNA load were recorded through a clinical case report form and measured according to routine care every 6 to 12 months | **Outcome definitions:**  Seroconversion: Evidence of HIV seroconversion in HIV-negative partner measured every 6 to 12 months. If HIV-negative partner found to become HIV-positive, venous blood sample from both partners was used to determine genetic relatedness of HIV-1 pol and env sequences.    Adherence: HIV-positive partner self-reported adherence to ART, rated from 0% to 100% over the previous month.  **Case ascertainment:** HIV-negative partner was asked to test for evidence of HIV seroconversion every 6 to 12 months  **Sampling methods:** NR for combined antigen/antibody test; venous blood sample for sequence testing  **Diagnostic tests:** Combined antigen/antibody test with subsequent sequence testing if positive  **Comments:** Results for MSM reported in Rodger 2019 | **HIV-related outcomes:**  Seroconversion: A total of 1/548 (0.18%) of the originally HIV-negative partners seroconverted, but there were no phylogenetically linked transmissions.  **Other related outcomes:**  Adherence >90%:   - Heterosexual men: 242/269 (93%) - Heterosexual women: 235/279 (94%) - MSM: 330/340 (97%)   **Adverse events:** NR |
| **Author**: Shan^75^  **Year:** 2023  **Data extractor:** DOS  **Reviewer:** CNS  **KQ(s):** 1, 3  **Study design:** Cross-sectional  **Study objective:** To explore the modality of online PEP services provision among Chinese MSM and to gain insight into the following: characteristics of online PEP recipients, uptake and outcomes of online PEP use, and factors of PrEP usage among those seeking PEP services through the internet.  **Limitations**: no major | **Population:**  N = 539 participants  **Setting:** Online  **Location:** China  **Study dates:** January 2020 – June 2021  **Matching:** None  **Inclusion criteria:** Participants who were over 18 years and had ever used PEP services via the ‘HeHealth’ internet medical platform and completed an anonymous online questionnaire. Each IP address was restricted to filling out only one questionnaire.  **Exclusion criteria:** Questionnaires with inconsistent information or core logic errors, and those of participants who completed the questionnaires within 30 seconds or submitted incomplete questionnaires were considered likely to be untruthful or not serious. | **Key Question 1:**  **Intervention group:** n = 493   - **ART regimen:** 28-day regimen of  1. Lamivudine, tenofovir disoproxil fumarate, and dolutegravir (3TC/TDF+DTG), n = 293 2. Emtricitabine, tenofovir disoproxil fumarate, and dolutegravir (FTC/TDF+DTG), n = 158 3. Emtricitabine, tenofovir alafenamide fumarate, and dolutegravir (FTC/TAF+DTG), n = 42   **Control group:** n = 34   - **ART regimen:** 28-day regimen of Emtricitabine, tenofovir disoproxil fumarate, and raltegravir (FTC/TDF/RAL)   **Key Question 3:**  **Intervention group:** n = 2  Initiated PEP after 72 hours   - **ART regimen:** NR   **Control group:** n = 537  Initiated PEP within 72 hours   - **ART regimen:** NR   **Exposure assignment or ascertainment:** Self-reported information on PEP regimen prescribed collected via online questionnaire. | **Outcome definitions:**  *Seroconversion:* Self-reported HIV seroconversion  *Adherence:* Self-reported completion of full 28-day course of medication  *Adverse events:* ND  **Case ascertainment:** Self-reported information on HIV status collected via online questionnaire.    **Sampling methods:** NR  **Diagnostic tests:** NR  **Comments:** None | **HIV-related outcomes:**  *Seroconversion:* No HIV seroconversions were observed among the 539 PEP users, 49/539 (9.1%) of whom reported continuing to engage in high-risk sexual behaviors while using PEP.  **Other related outcomes:**  *Adherence:* 523/539 (97.0%)  *Initiated PEP request >72 hours after exposure:* 2/539 (0.4%)  **Adverse events:** No serious adverse drug reactions following the intake of PEP drugs were observed for the surveyed participants. |
| **Author**: Shintani^88^  **Year:** 2020  **Data extractor:** CNS  **Reviewer:** DOS  **KQ(s):** 4  **Study design:** Case series  **Study objective:** To report on the utilization and clinical outcomes of PEP among dental staff after being potentially exposed to HIV during dental treatment.  **Limitations**: no major | **Population:** N = 5  **Setting:** University hospital  **Location:** Japan  **Study dates:** 2007 – 2018  **Matching:** NA  **Inclusion criteria:** Cases of accidental occupational exposure to HIV that had occurred in the dental departments.  **Exclusion criteria:** NR | **Intervention group:** n = 5  Dentists exposed to HIV-positive source patient with viral load of <20 copies/ml   - **ART regimen:** 1 dentist was on raltegravir + tenofovir/emtricitabine for four weeks and 1 dentist was on raltegravir + tenofovir alafenamide/ emtricitabine for four weeks - **Viral load of source patient:** 4 had viral loads of <20 copies/ml for 6 months or more and 1 had a viral load of <20 copies/ml for 2 months.   **Control group:** n = NA   - **ART regimen:** NA - **Viral load of source patient:** NA   **Exposure assignment or ascertainment:** Plasma HIV viral load and period of undetectable HIV viral load were examined for source patients. It is standard to measure once every 2-3 months, however, in some patients it is measured once a month. | **Outcome definitions:**  Seroconversion: HIV-positive test at 4 weeks or 12 weeks after exposure  **Case ascertainment:** HCP were followed up at 4 weeks and 12 weeks  **Sampling methods:** NR  **Diagnostic tests:** Fourth generation HIV Ag/Ab combination immunoassays  **Comments:** None | **HIV-related outcomes:**  Seroconversion: There were 0/5 (0%) exposed HCP who seroconverted up to 12 weeks after the exposure.  **Other related outcomes:**  Adherence: 2/2 (100%)  **Adverse events:** NR |
| **Author**: Sonder^80^  **Year:** 2007  **Data extractor:** DOS  **Reviewer:** CNS  **KQ(s):** 3  **Study design:** Cohort  **Study objective:** To evaluate trends in HIV PEP requests after sexual exposure, outcome of HIV testing of sources, PEP indications, completion of PEP courses, compliance with and the outcome of follow-up HIV tests.  **Limitations**: no major | **Population:** N = 169  **Setting:** Hospitals, Municipal Health Service  **Location:** The Netherlands  **Study dates:** January 1, 2000 – December 31, 2004  **Matching:** None  **Inclusion criteria:** All persons requesting PEP after sexual possible exposure (including rape victims) to HIV at a hospital who are then referred to Municipal Health Service for treatment and follow-up.  **Exclusion criteria:** PEP was not prescribed. | **Intervention group:** n ≥ 1 (1%)   - **ART regimen:** Standard combination therapy includes 200 mg nevirapine as a single dose followed by a 28-day course of 300/150 mg zidovudine/lamivudine twice a day and 1,250 mg nelfinavir twice a day - **Interval between HIV exposure and PEP initiation:** >72 hours   **Control group:** n ≥ 157 (94%)   - **ART regimen:** Standard combination therapy includes 200 mg nevirapine as a single dose followed by a 28-day course of 300/150 mg zidovudine/lamivudine twice a day and 1,250 mg nelfinavir twice a day - **Interval between HIV exposure and PEP initiation:** ≤72 hours   **Exposure assignment or ascertainment:** All patient data are entered in a Municipal Health Service database | **Outcome definitions:**  *Seroconversion:* Testing positive for HIV at three or six months  **Case ascertainment:** Patients were followed up at three and six months for HIV testing  **Sampling methods:** NR  **Diagnostic tests:** NR  **Comments:** None | **HIV-related outcomes:**  *Seroconversion:* Among MSM, 128/151 (85%) patients were tested at three months and 113/151 (75%) were tested at six months; the remaining did not comply. Everyone tested negative during follow-up except for one MSM who tested negative at three month and positive at six months follow-up. The patient reported receptive anal sexual exposure without ejaculation with a known HIV-positive source whose viral load had recent been tested as ‘undetectable.’ HIV PEP was started 5 hours after that exposure and the course was completed. After being informed that this exposure was unlikely to have cause infection found at six months, the patient admitted to unsafe contacts after completing PEP course.  **Other related outcomes:**  *PEP Completion:* 119/140 (85%)  **Adverse events:** NR |
| **Author**: Taylor^65^  **Year:** 2015  **Data extractor:** DOS  **Reviewer:** CNS  **KQ(s):** 5  **Study design: :** Diagnostic accuracy **Study objective:** To use knowledge of the eclipse period and data from commercial and literature-reported seroconversion panels to calculate the window periods for third- and fourth-generation HIV tests and to provide a table reflecting the probability of a negative test during the window period among known HIV-infected individuals.  **Limitations**: no major | **Population:**  N = 1,241 assays  **Setting:** Laboratory  **Location:** NR  **Study dates:** April 1981 – 2010  **Matching:** NA  **Inclusion criteria:** Publicly available data sheets from HIV-positive seroconversion reference panels that provided a third- or fourth-generation test result and a PCR test result and are currently in use for HIV diagnosis. Data from manuscripts with seroconversion data not already included in the publicly available data sheets.  **Exclusion criteria:** Results of rapid point-of-care tests, assays that did not have both a PCR and third- or fourth-generation test result, the assay was no longer in routine use, the donor did not seroconvert prior to cessation of donating plasma, the length of time between blood draws resulted in the PCR being positive on the same day as the antibody test, and duplicate records. | **Intervention group:** n = 660 assays   - **ART regimen:** NR - **HIV test used:** Fourth-generation tests     **Control group:** n = 581 assays   - **ART regimen:** NR - **HIV test used:** Third-generation tests     **Exposure assignment or ascertainment:** NR | **Outcome definitions:**  *Duration to detection:* Number of days from positive PCR/NAAT test to the first positive third- or fourth-generation test for each panel was calculated and added to an estimated eclipse period to represent number of days from infection to first positive third- or fourth-generation results.  *Probability of a false-negative:* Probability of a false-negative third- and/or fourth-generation EIA HIV test at various time intervals after HIV infection  **Case ascertainment:** Each HIV-positive seroconversion reference panel represented data from one HIV-infected individual. Manuscripts with data not included in reference panels reported on seroconversions.  **Sampling methods:** Blood draw  **Diagnostic tests:** Third-generation antibody and fourth-generation antigen/antibody  **Comments:** Current fourth-generation assays have improved since 2006. | **HIV-related outcomes:**  Duration to detection, median days (IQR):   - Fourth-generation tests: 18 (IQR: 16 to 24) - Third-generation tests: 22 (IQR: 19 to 25)   Duration to detection, mean days (SD):   - Fourth-generation tests: 20.45 (7.0) - Third-generation tests: 25.04 (13.1)   Probability of a false-negative: The probability of a false-negative result is 0.01 at 85 days post-exposure for third-generation tests (99% CI: 0.002 to 0.022) and 42 days for fourth-generation tests (99% CI: 0.05 to 0.027).  **Other related outcomes:** NR  **Adverse events:** NR |
| **Author**: Wu^73^  **Year:** 2021  **Data extractor:** CNS  **Reviewer:** ECS/DOS    **KQ(s):** 1  **Study design:** Cohort study  **Study objective:** To describe the efficacy of nPEP through medication adherence, follow-up retention, and HIV seroconversion among men who have sex with men (MSM) consulting for nPEP.  **Limitations**: no major | **Population:**  N = 112  **Setting:** Community/ outpatient  **Location:** China  **Study dates:** September 2017 – December 2019  **Matching:** None  **Inclusion criteria:** MSM ≥ 18 years old who tested HIV-negative, provided informed consent, and lived within the two study cities. Exposures that may warrant nPEP include having sex without condoms (including homosexual and heterosexual behaviors), sharing needles with HIV-infected drug users, exposure to blood, semen, genital secretions, blood-stained saliva, and wound exudate splashing to eye/nose/oral cavity/damaged mucosa.  **Exclusion criteria:** If the source is established to be HIV negative, if exposures to bodily fluids do not pose a significant risk (tears, non-blood stained saliva, urine, and sweat), or if the exposure time exceeds 72 hours. | **Intervention group:**  Intervention group 1, n = 15  Intervention group 2, n= 93  Participants who showed lab testing reports during follow-up visits obtained RMB 100 ($14.49) for transportation. Participants were reminded to adhere to their regimen and return for HIV testing through phone calls and social media. The first peer educator who received the patient would accompany that person to referral service to ART clinics and assist doctors in offering follow-up services (adherence education, adverse effect monitoring, psychological support, follow-up testing, and 24/7 consultation).   - **ART regimen of intervention group 1:** Tenofovir fumarate/emtricitabine (TDF/FTC) 300/ 200mg once daily + dolutegravir (DTG) 50mg once daily for 28 days - **ART regimen of intervention group 2:** Abacavir/lamivudine/dolutegravir (ABC/3TC/DTG) 50/600/300 mg combination tablet once daily for 28 days     **Control group:** n = 4  Participants who showed lab testing reports during follow-up visits obtained RMB 100 ($14.49) for transportation. Participants were reminded to adhere to their regimen and return for HIV testing through phone calls and social media. The first peer educator who received the patient would accompany that person to referral service to ART clinics and assist doctors in offering follow-up services (adherence education, adverse effect monitoring, psychological support, follow-up testing, and 24/7 consultation).   - **ART regimen:** Tenofovir fumarate/emtricitabine (TDF/FTC) 300/ 200mg once daily + raltegravir (RAL) 400mg twice daily for 28 days     **Exposure assignment or ascertainment:** Participants chose their preferred regimen and data were extracted from clinical records | **Outcome definitions:**  Seroconversion: HIV positive test at follow-up testing    Adverse events: Included mild gastrointestinal, neurologic disorders, and musculoskeletal pain  **Case ascertainment:**At baseline, patients were tested for HIV antibodies/antigens, complete blood count, and renal/liver function. Follow-up testing was completed by repeating HIV serology at week 4, week 6, and 3 months after exposure.  **Sampling methods:** NR  **Diagnostic tests:** Fourth generation antibody/antigen combo assay, and participants testing positive for HIV were referred to the CDC for western-blot, CD4 count, and viral load tests  **Comments:** None | **HIV-related outcomes:**  Seroconversion:  None of the patients seroconverted. However, one case that tested negative 6 weeks after exposure discontinued medication two weeks later and was confirmed positive on day 82 after ongoing high-risk behaviors including unprotected anal intercourse and oral penetration.  **Other related outcomes:** NR    **Adverse events:**   - DTG-based regimens: 7/107 (6.5%) - RAL-based regimen: AE: 1/4 (25.0%)   TDF/FTC+DTG:   - AE: 2/15 (13.3%) - No AE: 13/15 (86.7%)     ABC/3TC/DTG:   - AE: 5/92 (5.4%) - No AE: 87/92 (94.6%)     TDF/FTC+RAL:   - AE: 1/4 (25.0%) - No AE: 3/4 (75.0%) |

## **I.2. Assessment of** **Bias for Individual Studies**

**Figure S2**. Internal Validity Assessments for individual studies across signaling prompts


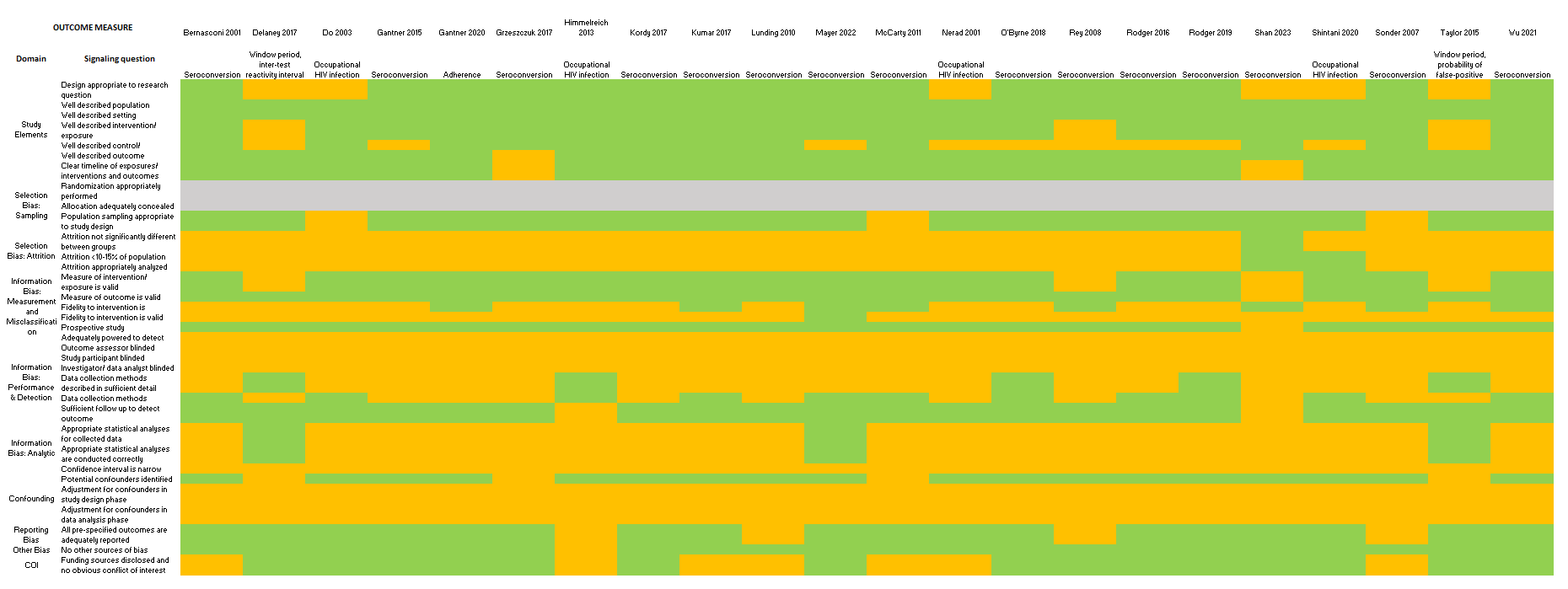


## **I.3. Summary of Expert Experience**

A diverse panel of experts were involved in the drafting and review of the recommendations in this guideline. Table S15 below contains a summary of workgroup and expert panel members’ experience with counseling, testing, or prescribing for healthcare worker (HCW) and patient exposures to HIV. While the combined years of experience, and number of healthcare workers counseled would be equivalent to many case reports, the average number of patients or HCW counseled per year of panel experience indicates the relative paucity of data there may be for some exposures during the <100 years of combined medical experience. The rarity of these exposures supports reliance upon expert experience for the clarification of specific recommendations. The number of HCW or patients who were counseled, tested, or treated by the workgroup and expert panel members are identified as case reports.

**Table S15.** Summary of expert experience informing the development of Good Practice Statements.

| **Question** |  | **Total Years** | **Years per expert (Mean)** |  |
| --- | --- | --- | --- | --- |
| How many years have you been practicing in your field? | | **101** | **20.5** | **--** |
|  | **Population** | **Case Reports** | **Case reports per expert (Mean)** | **Case reports per year of experience** |
| How many patients or healthcare workers with exposures to HIV have you counseled or prescribed PEP? | Patients: | **25** | **5** | **<1** |
|  | HCW: | **870** | **174** | **<9** |
| How many patients or healthcare workers have you counseled or managed who initiated PEP >24 hours after a single exposure or missed doses? | Patients: | **15** | **<8** | **<1** |
|  | HCW: | **<80** | **<28** | **1 – 2** |
| How many patients infected with HIV have you treated? | | **<1,650** | **<330** |  |
| How many patients or healthcare workers among the following populations have you prescribed the alternative Darunavir-based PEP regimens: | | | | |
| Pregnant | Patients: | **0** | 0 | 0 |
|  | HCW: | **1** | **1** | **<1** |
| Moderate or Severe renal dysfunction | Patients: | **0** | **0** | **0** |
|  | HCW: | **1** | **1** | **<1** |
| Hepatic Impairment | Patients: | **0** | **0** | **0** |
|  | HCW: | **1** | **1** | **<1** |
| None of the above conditions | Patients: | **0** | **0** | **0** |
|  | HCW: | **2** | **2** | **<1** |
| How many patients or healthcare workers with exposures to HIV, that later developed HIV infection, have you counseled or managed since PEP was made available? | Patients: | **0** | **0** | **0** |
|  | HCW: | **5** | **5** | **<1** |

#

# **J. Search Strategies**

**Table S16.** Literature search strategies and results by database

| **Database** | **Strategy** | **Records**  08/24/2022 | **Records**  06/15/2023 |
| --- | --- | --- | --- |
| **Medline**  **(OVID)**  **1946-** | Exp HIV Infections/ OR (HIV* OR human immunodeficiency virus*).ti,ab,kf.  AND  exp Post-Exposure Prophylaxis/ OR (Post-exposure* OR postexposure* OR PEP OR oPEP).ti,ab,kf. OR Exp Occupational exposure/ OR Needlestick Injuries/ OR ((occupational ADJ5 expos*) OR (healthcare* ADJ5 expos*) OR (health care* ADJ5 expos*) OR (worker* ADJ5 expos*) OR occupational hazard* OR occupational disease* OR occupational injur* OR (percutaneous ADJ5 expos*) OR (membrane ADJ5 expos*) OR needlestick injur* OR needle stick injur*).ti,ab,kf.  AND  Exp drug therapy/ OR exp Anti-Retroviral Agents/ OR Early Medical Intervention/ OR dt.fs OR (dolutegravir OR bictegravir* OR ART OR ARV OR antiretroviral* OR anti-retroviral* OR antiretrovirus* OR anti-retrovirus* OR DTG-3TC-ABC OR DTG-3TC OR BIC-TAF-3TC OR Raltegravir OR Truvada OR cabotegravir* OR CAB-RPV OR administer* OR administration OR antigen antibody test* OR ag ab test* OR breakthrough OR break-through OR (treatment ADJ5 failure*) OR (therap* ADJ5 failure*) OR regimen* OR medical intervention*).ti,ab,kf,hw.  Update (202208* OR 202209* OR 202210* OR 202211* OR 202212* OR 2023*).dt,ed | 2275 | 74 |
| **Embase**  **(OVID)**  **1974-** | Exp Human immunodeficiency virus infection/ OR (HIV* OR human immunodeficiency virus*).ti,ab,kf.  AND  exp Post-Exposure Prophylaxis/ OR (Post-exposure* OR postexposure* OR PEP OR oPEP).ti,ab,kf. OR Exp Occupational exposure/ OR Needlestick Injury/ OR ((occupational ADJ5 expos*) OR (healthcare* ADJ5 expos*) OR (health care* ADJ5 expos*) OR (worker* ADJ5 expos*) OR occupational hazard* OR occupational disease* OR occupational injur* OR (percutaneous ADJ5 expos*) OR (membrane ADJ5 expos*) OR needlestick injur* OR needle stick injur*).ti,ab,kf.  AND  Exp drug therapy/ OR exp Antiretrovirus agent/ OR early intervention/ OR dt.fs OR (dolutegravir OR bictegravir* OR ART OR ARV OR antiretroviral* OR anti-retroviral* OR antiretrovirus* OR anti-retrovirus* OR DTG-3TC-ABC OR DTG-3TC OR BIC-TAF-3TC OR Raltegravir OR Truvada OR cabotegravir* OR CAB-RPV OR administer* OR administration OR antigen antibody test* OR ag ab test* OR breakthrough OR break-through OR (treatment ADJ5 failure*) OR (therap* ADJ5 failure*) OR regimen* OR medical intervention*).ti,ab,kf,hw.  NOT  pubmed/medline  NOT  Conference abstracts  Update (202208* OR 202209* OR 202210* OR 202211* OR 202212* OR 2023*).dc,em | 2377  -  duplicates  =1417  unique items | 169  -  duplicates  =127  unique items |
| **Global Health**  **(OVID)**  **1910-** | HIV* OR human immunodeficiency virus*  AND  (Post-exposure* OR postexposure* OR PEP OR oPEP OR (occupational ADJ5 expos*) OR (healthcare* ADJ5 expos*) OR (health care* ADJ5 expos*) OR (worker* ADJ5 expos*) OR occupational hazard* OR occupational disease* OR occupational injur* OR (percutaneous ADJ5 expos*) OR (membrane ADJ5 expos*) OR needlestick injur* OR needle stick injur*).ti,ab,sh.  AND  (drug therapy OR Antiretrovirus agent OR early intervention OR dolutegravir OR bictegravir* OR ART OR ARV OR antiretroviral* OR anti-retroviral* OR antiretrovirus* OR anti-retrovirus* OR DTG-3TC-ABC OR DTG-3TC OR BIC-TAF-3TC OR Raltegravir OR Truvada OR cabotegravir* OR CAB-RPV OR administer* OR administration OR antigen antibody test* OR ag ab test* OR breakthrough OR break-through OR (treatment ADJ5 failure*) OR (therap* ADJ5 failure*) OR regimen* OR medical intervention*).ti,ab,sh.  Update (202208* OR 202209* OR 202210* OR 202211* OR 202212* OR 2023*).up | 949  -  duplicates  =385  unique items | 56  -  duplicates  =38  unique items |
| **Cochrane Library** | [mh "HIV Infections"] OR (HIV* OR "human immunodeficiency virus*"):ti,ab  AND  [mh "Post-Exposure Prophylaxis"] OR [mh "Occupational exposure"] OR [mh "Needlestick Injuries"] OR ("Post-exposure*" OR postexposure* OR PEP OR oPEP OR (occupational NEAR/5 expos*) OR (healthcare* NEAR/5 expos*) OR ("health care*" NEAR/5 expos*) OR (worker* NEAR/5 expos*) OR "occupational hazard*" OR "occupational disease*" OR "occupational injur*" OR (percutaneous NEAR/5 expos*) OR (membrane NEAR/5 expos*) OR "needlestick injur*" OR "needle stick injur*"):ti,ab  AND  [mh "drug therapy"] OR [mh "Anti-Retroviral Agents"] OR [mh "Early Medical Intervention"] OR (dolutegravir OR bictegravir* OR ART OR ARV OR antiretroviral* OR anti-retroviral* OR antiretrovirus* OR anti-retrovirus* OR DTG-3TC-ABC OR DTG-3TC OR BIC-TAF-3TC OR Raltegravir OR Truvada OR cabotegravir* OR CAB-RPV OR administer* OR administration OR "antigen antibody test*" OR "ag ab test*" OR breakthrough OR break-through OR (treatment NEAR/5 failure*) OR (therap* NEAR/5 failure*) OR regimen* OR "medical intervention*"):ti,ab  Update Limit to August 2022 - | 132  -  duplicates  =72  unique items | 4  -  duplicates  =3  unique items |
| **CINAHL**  **(EBSCOHost)** | (MH "HIV Infections+") OR (HIV* OR "human immunodeficiency virus*")  AND  (MH "Post-Exposure Prophylaxis+") OR (MH "Occupational exposure") OR (MH "Needlestick Injuries") OR ("Post-exposure*" OR postexposure* OR PEP OR oPEP OR (occupational N5 expos*) OR (healthcare* N5 expos*) OR ("health care*" N5 expos*) OR (worker* N5 expos*) OR "occupational hazard*" OR "occupational disease*" OR "occupational injur*" OR (percutaneous N5 expos*) OR (membrane N5 expos*) OR "needlestick injur*" OR "needle stick injur*")  AND  (MH "drug therapy+") OR (MH "Anti-Retroviral Agents+") OR (MH "Early Medical Intervention") OR (dolutegravir OR bictegravir* OR ART OR ARV OR antiretroviral* OR anti-retroviral* OR antiretrovirus* OR anti-retrovirus* OR DTG-3TC-ABC OR DTG-3TC OR BIC-TAF-3TC OR Raltegravir OR Truvada OR cabotegravir* OR CAB-RPV OR administer* OR administration OR "antigen antibody test*" OR "ag ab test*" OR breakthrough OR break-through OR (treatment N5 failure*) OR (therap* N5 failure*) OR regimen* OR "medical intervention*")  Exclude Medline Records  Update Limit to August 2022 - | 376  -  duplicates  = 284  unique items | 15  -  duplicates  =5  unique items |
| **Scopus** | TITLE-ABS-KEY(HIV* OR "human immunodeficiency virus*") AND TITLE-ABS-KEY("Post-exposure*" OR postexposure* OR PEP OR oPEP OR (occupational W/5 expos*) OR (healthcare* W/5 expos*) OR ("health care*" W/5 expos*) OR (worker* W/5 expos*) OR "occupational hazard*" OR "occupational disease*" OR "occupational injur*" OR (percutaneous W/5 expos*) OR (membrane W/5 expos*) OR "needlestick injur*" OR "needle stick injur*") AND TITLE-ABS-KEY("drug therap*" OR dolutegravir OR bictegravir* OR ART OR ARV OR antiretroviral* OR anti-retroviral* OR antiretrovirus* OR anti-retrovirus* OR DTG-3TC-ABC OR DTG-3TC OR BIC-TAF-3TC OR Raltegravir OR Truvada OR cabotegravir* OR CAB-RPV OR "antigen antibody test*" OR "ag ab test*" OR breakthrough OR break-through OR (treatment W/5 failure*) OR (therap* W/5 failure*) OR regimen* OR "medical intervention*") AND NOT INDEX(medline) | 456  -  duplicates  = 112  unique items | 92  -  duplicates  =33  unique items |

# **K. All References in the Main Document and Appendix**

1. Kuhar DT, Henderson DK, Struble KA, et al. Updated US Public Health Service guidelines for the management of occupational exposures to human immunodeficiency virus and recommendations for postexposure prophylaxis. *Infect Control Hosp Epidemiol*. Sep 2013;34(9):875-92. doi:<http://doi.org/10.1086/672271>.

2. Kuhar DT, Henderson DK, Struble KA, et al. Updated U.S. Public Health Service guidelines for the management of occupational exposures to HIV and recommendations for postexposure prophylaxis. Pamphlet (or booklet). Updated 25/2013 Update (May 23, 2018). <https://stacks.cdc.gov/view/cdc/20711>

3. The Healthcare Infection Control Practices Advisory Committee. Update to the Centers for Disease Control and Prevention and the Healthcare Infection Control Practices Advisory Committee Recommendation Categorization Scheme for Infection Control and Prevention Guideline Recommendations. Accessed March 20, 2025. <https://www.cdc.gov/hicpac/media/pdfs/recommendation-scheme-update-508.pdf>

4. Guyatt GH, Oxman AD, Vist GE, et al. GRADE: an emerging consensus on rating quality of evidence and strength of recommendations. *BMJ*. Apr 26 2008;336(7650):924-6. doi:<http://doi.org/10.1136/bmj.39489.470347.AD>.

5. Dewidar O, Lotfi T, Langendam MW, et al. Good or best practice statements: proposal for the operationalisation and implementation of GRADE guidance. *BMJ Evid Based Med*. Jun 2023;28(3):189-196. doi:<http://doi.org/10.1136/bmjebm-2022-111962>.

6. Siegel JD, Rhinehart E, Jackson M, Chiarello L, and Health Care Infection Control Practices Advisory Committee. 2007 Guideline for Isolation Precautions: Preventing Transmission of Infectious Agents in Health Care Settings. *Am J Infect Control*. Dec 2007;35(10 Suppl 2):S65-164. doi:<https://doi.org/10.1016/j.ajic.2007.10.007>.

7. Occupational Safety and Health Administration. Occupational Exposure to Bloodborne Pathogens Standard (1910.1030). Occupational Safety and Health Administration. Accessed March 20, 2025. <https://www.osha.gov/laws-regs/regulations/standardnumber/1910/1910.1030>

8. Schillie S, Murphy TV, Sawyer M, et al. CDC guidance for evaluating health-care personnel for hepatitis B virus protection and for administering postexposure management. *MMWR Recomm Rep*. Dec 20 2013;62(RR-10):1-19.

9. Moorman AC, de Perio MA, Goldschmidt R, et al. Testing and Clinical Management of Health Care Personnel Potentially Exposed to Hepatitis C Virus - CDC Guidance, United States, 2020. *MMWR Recomm Rep*. Jul 24 2020;69(6):1-8. doi:<http://doi.org/10.15585/mmwr.rr6906a1>

10. Tanner MR, O'Shea JG, Byrd KM, et al. Antiretroviral Postexposure Prophylaxis After Sexual, Injection Drug Use, or Other Nonoccupational Exposure to HIV - CDC Recommendations, United States, 2025. *MMWR Recomm Rep*. May 8 2025;74(1):1-56. doi:10.15585/mmwr.rr7401a1

11. Panel on Antiretroviral Therapy and Medical Management of Children Living with HIV. Guidelines for the Use of Antiretroviral Agents in Pediatric HIV Infection. U.S. Department of Health and Human Services. Accessed March 20, 2025. <https://clinicalinfo.hiv.gov/en/guidelines/pediatric-arv>

12. Panel on Treatment of HIV During Pregnancy and Prevention of Perinatal Transmission. Recommendations for the Use of Antiretroviral Drugs During Pregnancy and Interventions to Reduce Perinatal HIV Transmission in the United States. U.S. Department of Health and Human Services, . Accessed March 20, 2025. <https://clinicalinfo.hiv.gov/en/guidelines/perinatal>

13. Page MJ, Moher D, Bossuyt PM, et al. PRISMA 2020 explanation and elaboration: updated guidance and exemplars for reporting systematic reviews. *BMJ*. Mar 29 2021;372:n160. doi:<http://doi.org/10.1136/bmj.n160>.

14. The Healthcare Infection Control Practices Advisory Committee. Record of the Proceedings of the August 22, 2023 Meeting. 2023:

15. U.S. Centers for Disease Control and Prevention. 7. Management of Potentially Infectious Exposures and Illnesses in Infection Control in Healthcare Personnel: Infrastructure and Routine Practices for Occupational Infection Prevention and Control Services (2019). . Accessed March 20, 2025. <https://www.cdc.gov/infectioncontrol/guidelines/healthcare-personnel/exposures.html>

16. Panel on Antiretroviral Guidelines for Adults and Adolescents. Guidelines for the Use of Antiretroviral Agents in Adults and Adolescents with HIV. Accessed March 20, 2025. <https://clinicalinfo.hiv.gov/en/guidelines/adult-and-adolescent-arv>.

17. U.S. Centers for Disease Control and Prevention. Appendix 2: Terminology, in Infection Control in Healthcare Personnel: Infrastructure and Routine Practices for Occupational Infection Prevention and Control Services (2019). Accessed March 20, 2025. <https://www.cdc.gov/infection-control/hcp/healthcare-personnel-infrastructure-routine-practices/terminology.html>

18. U.S. Centers for Disease Control and Prevention. How HIV Spreads, HIV. Accessed March 20, 2025. <https://www.cdc.gov/hiv/causes/index.html>

19. Bell DM. Occupational risk of human immunodeficiency virus infection in healthcare workers: an overview. *The American journal of medicine*. 1997;102(5):9-15.

20. Meechan PJ, Potts J. Biosafety in microbiological and biomedical laboratories. Accessed March 20, 2025. <https://stacks.cdc.gov/view/cdc/97733>

21. Wahn V, Kramer HH, Voit T, Bruster HT, Scrampical B, Scheid A. Horizontal transmission of HIV infection between two siblings. *Lancet*. Sep 20 1986;2(8508):694. doi:<http://doi.org/10.1016/s0140-6736(86)90209-6>.

22. Anonymous. Transmission of HIV by human bite. *Lancet*. Aug 29 1987;2(8557):522.

23. Richman KM, Rickman LS. The potential for transmission of human immunodeficiency virus through human bites. *J Acquir Immune Defic Syndr (1988)*. Apr 1993;6(4):402-6.

24. Vidmar L, Poljak M, Tomazic J, Seme K, Klavs I. Transmission of HIV-1 by human bite. *Lancet*. Jun 22 1996;347(9017):1762. doi:<http://doi.org/10.1016/s0140-6736(96)90838-7>.

25. Deshpande AK, Jadhav SK, Bandivdekar AH. Possible transmission of HIV Infection due to human bite. *AIDS Res Ther*. Mar 31 2011;8:16. doi:<http://doi.org/10.1186/1742-6405-8-16>.

26. Andreo SM, Barra LA, Costa LJ, Sucupira MC, Souza IE, Diaz RS. HIV type 1 transmission by human bite. *AIDS Res Hum Retroviruses*. Apr 2004;20(4):349-50. doi:<http://doi.org/10.1089/088922204323048087>.

27. Schurmann D, Hoffmann C, Stegemann MS, Ruwwe-Glosenkamp C, Gurtler L. HIV transmission by human bite: a case report and review of the literature-implications for post-exposure prophylaxis. *Infection*. Dec 2020;48(6):949-954. doi:<http://doi.org/10.1007/s15010-020-01477-6>.

28. U.S. Centers for Disease Control and Prevention. Public Health Service guidelines for the management of health-care worker exposures to HIV and recommendations for postexposure prophylaxis. *MMWR Recomm Rep*. 1998;47(RR-7):1-33.

29. U.S. Centers for Disease Control and Prevention. Updated U.S. Public Health Service Guidelines for the Management of Occupational Exposures to HBV, HCV, and HIV and Recommendations for Postexposure Prophylaxis. *MMWR Recomm Rep*. Jun 29 2001;50(RR-11):1-52.

30. Baggaley RF, Boily MC, White RG, Alary M. Risk of HIV-1 transmission for parenteral exposure and blood transfusion: a systematic review and meta-analysis. *AIDS*. Apr 4 2006;20(6):805-12. doi:<http://doi.org/10.1097/01.aids.0000218543.46963.6d>.

31. Ippolito G, Puro V, De Carli G. The risk of occupational human immunodeficiency virus infection in health care workers. Italian Multicenter Study. The Italian Study Group on Occupational Risk of HIV infection. *Arch Intern Med*. Jun 28 1993;153(12):1451-8.

32. Cardo DM, Culver DH, Ciesielski CA, et al. A case-control study of HIV seroconversion in health care workers after percutaneous exposure. Centers for Disease Control and Prevention Needlestick Surveillance Group. *N Engl J Med*. Nov 20 1997;337(21):1485-90. doi:<http://doi.org/10.1056/NEJM199711203372101>.

33. Mast ST, Woolwine JD, Gerberding JL. Efficacy of gloves in reducing blood volumes transferred during simulated needlestick injury. *J Infect Dis*. Dec 1993;168(6):1589-92. doi:<http://doi.org/10.1093/infdis/168.6.1589>.

34. Shih CC, Kaneshima H, Rabin L, et al. Postexposure prophylaxis with zidovudine suppresses human immunodeficiency virus type 1 infection in SCID-hu mice in a time-dependent manner. *J Infect Dis*. Mar 1991;163(3):625-7. doi:<http://doi.org/10.1093/infdis/163.3.625>.

35. Tsai CC, Emau P, Follis KE, et al. Effectiveness of postinoculation (R)-9-(2-phosphonylmethoxypropyl) adenine treatment for prevention of persistent simian immunodeficiency virus SIVmne infection depends critically on timing of initiation and duration of treatment. *J Virol*. May 1998;72(5):4265-73. doi:<http://doi.org/10.1128/JVI.72.5.4265-4273.1998>.

36. Whitney JB, Hill AL, Sanisetty S, et al. Rapid seeding of the viral reservoir prior to SIV viraemia in rhesus monkeys. *Nature*. Aug 7 2014;512(7512):74-7. doi:<http://doi.org/10.1038/nature13594>.

37. Whitney JB, Lim SY, Osuna CE, et al. Prevention of SIVmac251 reservoir seeding in rhesus monkeys by early antiretroviral therapy. *Nat Commun*. Dec 21 2018;9(1):5429. doi:<http://doi.org/10.1038/s41467-018-07881-9>.

38. Massud I, Ruone S, Zlotorzynska M, et al. Single oral dose for HIV pre or post-exposure prophylaxis: user desirability and biological efficacy in macaques. *EBioMedicine*. Aug 2020;58:102894. doi:<http://doi.org/10.1016/j.ebiom.2020.102894>.

39. Otten RA, Smith DK, Adams DR, et al. Efficacy of postexposure prophylaxis after intravaginal exposure of pig-tailed macaques to a human-derived retrovirus (human immunodeficiency virus type 2). *J Virol*. Oct 2000;74(20):9771-5. doi:<http://doi.org/10.1128/jvi.74.20.9771-9775.2000>.

40. Tsai CC, Follis KE, Sabo A, et al. Prevention of SIV infection in macaques by (R)-9-(2-phosphonylmethoxypropyl)adenine. *Science*. Nov 17 1995;270(5239):1197-9. doi:<http://doi.org/10.1126/science.270.5239.1197>.

41. Henderson DK. Human immunodeficiency virus in health care settings. *Mandell, Douglas, and Bennett's principles and practice of infectious diseases*. Elsevier; 2015:3361-3375. e4.

42. U.S. Food and Drug Administration. Reporting Serious Problems to FDA. U.S. Food and Drug Administration,. Accessed March 20, 2025. <https://www.fda.gov/safety/medwatch-fda-safety-information-and-adverse-event-reporting-program/reporting-serious-problems-fda>

43. Eshleman SH, Fogel JM, Piwowar-Manning E, et al. Characterization of Human Immunodeficiency Virus (HIV) Infections in Women Who Received Injectable Cabotegravir or Tenofovir Disoproxil Fumarate/Emtricitabine for HIV Prevention: HPTN 084. *J Infect Dis*. May 16 2022;225(10):1741-1749. doi:10.1093/infdis/jiab576

44. Srinivasan P, Zhang J, Edwards T, et al. Efficacy of long acting cabotegravir and rilpivirine for PEP in a macaque model of RT SHIV infection. Abstract 1132. 2024:

45. U.S. Centers for Disease Control and Prevention. Serious Adverse Events Attributed to Nevirapine Regimens for Postexposure Prophylaxis After HIV Exposures. *MMWR Morb Mortal Wkly Rep*. 2001;49(51):1153-6.

46. Beltrami EM, Cheingsong R, Heneine WM, et al. Antiretroviral drug resistance in human immunodeficiency virus-infected source patients for occupational exposures to healthcare workers. *Infect Control Hosp Epidemiol*. Oct 2003;24(10):724-30. doi:<http://doi.org/10.1086/502120>.

47. Hawkins DA, Asboe D, Barlow K, Evans B. Seroconversion to HIV-1 following a needlestick injury despite combination post-exposure prophylaxis. *J Infect*. Jul 2001;43(1):12-5. doi:<http://doi.org/10.1053/jinf.2001.0811>.

48. Beltrami EM, Luo CC, de la Torre N, Cardo DM. Transmission of drug-resistant HIV after an occupational exposure despite postexposure prophylaxis with a combination drug regimen. *Infect Control Hosp Epidemiol*. Jun 2002;23(6):345-8. doi:<http://doi.org/10.1086/502065>.

49. Perdue B, Wolfe Rufael D, Mellors J, Quinn T, Margolick J. HIV-1 transmission by a needle-stick injury despite rapid initiation of four-drug postexposure prophylaxis. . 1999:

50. Lockman S, Creek T. Acute maternal HIV infection during pregnancy and breast-feeding: substantial risk to infants. *J Infect Dis*. Sep 1 2009;200(5):667-9. doi:<http://doi.org/10.1086/605124>.

51. U.S. Centers for Disease Control and Prevention. Infection Control in Healthcare Personnel. Epidemiology and Control of Selected Infections (2024). Accessed March 20, 2025. <https://www.cdc.gov/infection-control/hcp/healthcare-personnel-epidemiology-control/index.html>

52. Mirochnick M, Thomas T, Capparelli E, et al. Antiretroviral concentrations in breast-feeding infants of mothers receiving highly active antiretroviral therapy. *Antimicrob Agents Chemother*. Mar 2009;53(3):1170-6. doi:<http://doi.org/10.1128/AAC.01117-08>.

53. Benaboud S, Pruvost A, Coffie PA, et al. Concentrations of tenofovir and emtricitabine in breast milk of HIV-1-infected women in Abidjan, Cote d'Ivoire, in the ANRS 12109 TEmAA Study, Step 2. *Antimicrob Agents Chemother*. Mar 2011;55(3):1315-7. doi:<http://doi.org/10.1128/AAC.00514-10>.

54. U.S. Public Health Service. Preexposure Prophylaxis for the Prevention of HIV Infection in the United States - 2021 Update. A Clinical Practice Guideline. U.S. Centers for Disease Control and Prevention,. <https://stacks.cdc.gov/view/cdc/112360>

55. Hodge D, Back DJ, Gibbons S, Khoo SH, Marzolini C. Pharmacokinetics and Drug-Drug Interactions of Long-Acting Intramuscular Cabotegravir and Rilpivirine. *Clin Pharmacokinet*. Jul 2021;60(7):835-853. doi:<http://doi.org/10.1007/s40262-021-01005-1>.

56. Panlilio AL, Sinkowitz-Cochran R, Grady MA, Cardo DM. Barries to and facilitators of implementing U.S. Public Health Services (PHS) guidelines on occupational exposure management by emergency physicians. . 2003:

57. Masciotra S, McDougal JS, Feldman J, Sprinkle P, Wesolowski L, Owen SM. Evaluation of an alternative HIV diagnostic algorithm using specimens from seroconversion panels and persons with established HIV infections. *J Clin Virol*. Dec 2011;52 Suppl 1:S17-22. doi:<http://doi.org/10.1016/j.jcv.2011.09.011>.

58. Masciotra S, Luo W, Youngpairoj AS, et al. Performance of the Alere Determine HIV-1/2 Ag/Ab Combo Rapid Test with specimens from HIV-1 seroconverters from the US and HIV-2 infected individuals from Ivory Coast. *J Clin Virol*. Dec 2013;58 Suppl 1:e54-8. doi:<http://doi.org/10.1016/j.jcv.2013.07.002>.

59. Cohen MS, Chen YQ, McCauley M, et al. Prevention of HIV-1 infection with early antiretroviral therapy. *N Engl J Med*. Aug 11 2011;365(6):493-505. doi:<http://doi.org/10.1056/NEJMoa1105243>.

60. Eye of the Needle Report. Surveillance of significant occupational exposures to bloodborne viruses in healthcare workers in the United Kingdom - update on seroconversions. February (Public Health England.) (2020).

61. Joyce MP, Kuhar D, Brooks JT. Notes from the field: occupationally acquired HIV infection among health care workers - United States, 1985-2013. *MMWR Morb Mortal Wkly Rep*. Jan 9 2015;63(53):1245-6.

62. Armstrong K, Gorden R, Santorella G. Occupational exposure of health care workers (HCWs) to human immunodeficiency virus (HIV): stress reactions and counseling interventions. *Soc Work Health Care*. 1995;21(3):61-80. doi:<http://doi.org/10.1300/J010v21n03_06>.

63. Meienberg F, Bucher HC, Sponagel L, Zinkernagel C, Gyr N, Battegay M. Anxiety in health care workers after exposure to potentially HIV-contaminated blood or body fluids. *Swiss Med Wkly*. Jun 15 2002;132(23-24):321-4. doi:<http://doi.org/10.4414/smw.2002.09988>.

64. Delaney KP, Hanson DL, Masciotra S, Ethridge SF, Wesolowski L, Owen SM. Time Until Emergence of HIV Test Reactivity Following Infection With HIV-1: Implications for Interpreting Test Results and Retesting After Exposure. *Clin Infect Dis*. Jan 1 2017;64(1):53-59. doi:<http://doi.org/10.1093/cid/ciw666>.

65. Taylor D, Durigon M, Davis H, et al. Probability of a false-negative HIV antibody test result during the window period: a tool for pre- and post-test counselling. *Int J STD AIDS*. Mar 2015;26(4):215-24. doi:<http://doi.org/10.1177/0956462414542987>.

66. U.S. Centers for Disease Control and Prevention. National HIV Surveillance System (NHSS). U.S. Centers for Disease Control and Prevention. Accessed March 20, 2025. <https://www.cdc.gov/hiv-data/nhss/index.html>

67. Henderson DK, Dembry LM, Sifri CD, et al. Management of healthcare personnel living with hepatitis B, hepatitis C, or human immunodeficiency virus in US healthcare institutions. *Infect Control Hosp Epidemiol*. Feb 2022;43(2):147-155. doi:<http://doi.org/10.1017/ice.2020.458>.

68. Hosseini M-S, Jahanshahlou F, Akbarzadeh MA, Zarei M, Vaez-Gharamaleki Y. Formulating research questions for evidence-based studies. *Journal of Medicine, Surgery, and Public Health*. 2024/04/01/ 2024;2:100046. doi:<https://doi.org/10.1016/j.glmedi.2023.100046>

69. Mustafa RA, Garcia CAC, Bhatt M, et al. GRADE notes: How to use GRADE when there is "no" evidence? A case study of the expert evidence approach. *J Clin Epidemiol*. Sep 2021;137:231-235. doi:<http://doi.org/10.1016/j.jclinepi.2021.02.026>

70. Davidson KW, Mangione CM, Barry MJ, et al. Collaboration and Shared Decision-Making Between Patients and Clinicians in Preventive Health Care Decisions and US Preventive Services Task Force Recommendations. *JAMA*. Mar 22 2022;327(12):1171-1176. doi:10.1001/jama.2022.3267

71. Gantner P, Allavena C, Duvivier C, et al. Post-exposure prophylaxis completion and condom use in the context of potential sexual exposure to HIV. *HIV Med*. Aug 2020;21(7):463-469. doi:10.1111/hiv.12880

72. Kumar T, Sampsel K, Stiell IG. Two, three, and four-drug regimens for HIV post-exposure prophylaxis in a North American sexual assault victim population. *Am J Emerg Med*. Dec 2017;35(12):1798-1803. doi:10.1016/j.ajem.2017.05.054

73. Wu Y, Zhu Q, Zhou Y, et al. Implementation of HIV non-occupational post-exposure prophylaxis for men who have sex with men in 2 cities of Southwestern China. *Medicine (Baltimore)*. Oct 29 2021;100(43):e27563. doi:10.1097/md.0000000000027563

74. Mayer KH, Gelman M, Holmes J, Kraft J, Melbourne K, Mimiaga MJ. Safety and Tolerability of Once Daily Coformulated Bictegravir, Emtricitabine, and Tenofovir Alafenamide for Postexposure Prophylaxis After Sexual Exposure. *J Acquir Immune Defic Syndr*. May 1 2022;90(1):27-32. doi:10.1097/qai.0000000000002912

75. Shan D, Xue H, Yu F, et al. Understanding the Uptake and Outcomes of Non-occupational Postexposure Prophylaxis Use Through an Online Medical Platform in China: Web-Based Cross-sectional Study. *J Med Internet Res*. 2023/5/19 2023;25:e42729. doi:10.2196/42729

76. Shih C-C, Kaneshima H, Rabin L, et al. Postexposure Prophylaxis with Zidovudine Suppresses Human Immunodeficiency Virus Type 1 Infection in SCID-hu Mice in a Time-Dependent Manner. *The Journal of Infectious Diseases*. 1991;163(3):625-627. doi:10.1093/infdis/163.3.625

77. Do AN, Ciesielski CA, Metler RP, Hammett TA, Li J, Fleming PL. Occupationally acquired human immunodeficiency virus (HIV) infection: national case surveillance data during 20 years of the HIV epidemic in the United States. *Infect Control Hosp Epidemiol*. Feb 2003;24(2):86-96. doi:10.1086/502178

78. McCarty EJ, Quah S, Maw R, Dinsmore WW, Emerson CR. Post-exposure prophylaxis following sexual exposure to HIV: a seven-year retrospective analysis in a regional centre. *Int J STD AIDS*. Jul 2011;22(7):407-8. doi:10.1258/ijsa.2009.009463

79. Rey D, Bendiane MK, Bouhnik AD, Almeda J, Moatti JP, Carrieri MP. Physicians' and patients' adherence to antiretroviral prophylaxis after sexual exposure to HIV: results from South-Eastern France. *AIDS Care*. May 2008;20(5):537-41. doi:10.1080/09540120701867198

80. Sonder GJ, van den Hoek A, Regez RM, et al. Trends in HIV postexposure prophylaxis prescription and compliance after sexual exposure in Amsterdam, 2000-2004. *Sex Transm Dis*. May 2007;34(5):288-93. doi:10.1097/01.olq.0000237838.43716.ee

81. Bernasconi E, Jost J, Ledergerber B, Hirschel B, Francioli P, Sudre P. Antiretroviral prophylaxis for community exposure to the human immunodeficiency virus in Switzerland, 1997-2000. *Swiss Med Wkly*. Jul 28 2001;131(29-30):433-7. doi:10.4414/smw.2001.09755

82. Himmelreich H, Rabenau HF, Rindermann M, et al. The management of needlestick injuries. *Dtsch Arztebl Int*. Feb 2013;110(5):61-7. doi:10.3238/arztebl.2013.0061

83. Kordy F, Petrich A, Read SE, Bitnun A. Childhood exposures to discarded needles and other objects potentially contaminated with blood-borne pathogens in Toronto, Canada. *Paediatr Child Health*. Oct 2017;22(7):372-376. doi:10.1093/pch/pxx110

84. Lunding S, Katzenstein TL, Kronborg G, et al. The Danish PEP registry: experience with the use of postexposure prophylaxis (PEP) following sexual exposure to HIV from 1998 to 2006. *Sex Transm Dis*. Jan 2010;37(1):49-52. doi:10.1097/OLQ.0b013e3181b6f284

85. Rodger AJ, Cambiano V, Bruun T, et al. Risk of HIV transmission through condomless sex in serodifferent gay couples with the HIV-positive partner taking suppressive antiretroviral therapy (PARTNER): final results of a multicentre, prospective, observational study. *Lancet*. Jun 15 2019;393(10189):2428-2438. doi:10.1016/s0140-6736(19)30418-0

86. Rodger AJ, Cambiano V, Bruun T, et al. Sexual Activity Without Condoms and Risk of HIV Transmission in Serodifferent Couples When the HIV-Positive Partner Is Using Suppressive Antiretroviral Therapy. *JAMA*. Jul 12 2016;316(2):171-81. doi:10.1001/jama.2016.5148

87. Grzeszczuk J, Wroblewska A, Firląg-Burkacka E, Kowalska JD. The characteristics of HIV serodiscordant couples consulted at the HIV Out-Patient Clinic in Warsaw. journal article. *HIV &amp; AIDS Review International Journal of HIV-Related Problems*. 2017;16(1):58-60. doi:10.5114/hivar.2017.66798

88. Shintani T, Iwata T, Okada M, et al. Clinical Outcomes of Post-exposure Prophylaxis following Occupational Exposure to Human Immunodeficiency Virus at Dental Departments of Hiroshima University Hospital. *Curr HIV Res*. 2020;18(6):475-479. doi:10.2174/1570162x18666200804151118

89. Nerad JL, Kessler HA. Hypercholesterolemia in a health care worker receiving thyroxine after postexposure prophylaxis for human immunodeficiency virus infection. *Clin Infect Dis*. Jun 1 2001;32(11):1635-6. doi:10.1086/320528

90. Public Health England, Significant Occupational Exposures Team. Eye of the Needle: Surveillance of significant occupational exposures to bloodborne viruses in healthcare workers in the United Kingdom - update on seroconversions. Accessed 2/21, 2023. <https://shbn.org.uk/wp-content/uploads/2021/02/Eye-of-the-Needle-Report-February-2020.pdf>

91. Gantner P, Treger M, De Miscault C, et al. Predictors of Standard Follow-Up Completion after Sexual Exposure to HIV: Five-Year Retrospective Analysis in a French HIV-Infection Care Center. *PLoS One*. 2015;10(12):e0145440. doi:10.1371/journal.pone.0145440

92. O'Byrne P, MacPherson P, Orser L. Nurse-Led HIV PEP Program Used by Men at High Risk for HIV Seroconversion. *J Assoc Nurses AIDS Care*. Jul-Aug 2018;29(4):550-559. doi:10.1016/j.jana.2018.02.004

93. Fiebig EW, Wright DJ, Rawal BD, et al. Dynamics of HIV viremia and antibody seroconversion in plasma donors: implications for diagnosis and staging of primary HIV infection. *Aids*. Sep 5 2003;17(13):1871-9. doi:10.1097/00002030-200309050-00005

94. Colby DJ, Trautmann L, Pinyakorn S, et al. Rapid HIV RNA rebound after antiretroviral treatment interruption in persons durably suppressed in Fiebig I acute HIV infection. *Nat Med*. Jul 2018;24(7):923-926. doi:10.1038/s41591-018-0026-6

95. Donnell D, Ramos E, Celum C, et al. The effect of oral preexposure prophylaxis on the progression of HIV-1 seroconversion. *Aids*. Sep 10 2017;31(14):2007-2016. doi:10.1097/qad.0000000000001577

96. Kroon E, Chottanapund S, Buranapraditkun S, et al. Paradoxically Greater Persistence of HIV RNA-Positive Cells in Lymphoid Tissue When ART Is Initiated in the Earliest Stage of Infection. *The Journal of Infectious Diseases*. 2022;225(12):2167-2175. doi:10.1093/infdis/jiac089

97. Rothenberger MK, Keele BF, Wietgrefe SW, et al. Large number of rebounding/founder HIV variants emerge from multifocal infection in lymphatic tissues after treatment interruption. *Proc Natl Acad Sci U S A*. Mar 10 2015;112(10):E1126-34. doi:10.1073/pnas.1414926112

98. Hurt CB, Nelson JAE, Hightow-Weidman LB, Miller WC. Selecting an HIV Test: A Narrative Review for Clinicians and Researchers. *Sex Transm Dis*. Dec 2017;44(12):739-746. doi:10.1097/olq.0000000000000719

99. Work Group 2 Diagnostics & Laboratory HIV & STI, Federal Office of Public Health of the Swiss Confederation. Reassessment of the Diagnostics Window Period for HIV Diagnostics. Accessed February 5, 2025. <https://www.bag.admin.ch/dam/bag/de/dokumente/mt/p-und-p/richtlinien-empfehlungen/eksg-rec-window-period-hiv-diagnosis.pdf.download.pdf/eksg-rec-window-period-hiv-diagnosis.pdf>

1. Limitations/ loss of confidence due to: 1) Indirectness: no studies were conducted in occupational populations; and 2) Precision: All four studies reported no events. Increase in confidence due to: All plausible confounding would increase the rate of events in either group. [↑](#footnote-ref-1)
2. Limitations/ loss of confidence due to: Indirectness: Populations are indirect [↑](#footnote-ref-2)
3. Limitations/ loss of confidence due to: Indirectness: Populations are indirect [↑](#footnote-ref-3)
4. Limitations/ loss of confidence due to: 1) Precision: small sample sizes and low number of events, and 2) Indirectness: four studies^4-7^ are conducted in non-occupational populations. [↑](#footnote-ref-4)
5. Limitations/ loss of confidence due to: Risk of Bias: one study^4^ assumed that all specimen used for analysis were infected with HIV-1 subtype B; other HIV subtypes may have different durations to detection. Both studies^3,4^ were conducted among commercially and publicly available tests. [↑](#footnote-ref-5)
6. Limitations/ loss of confidence due to: 1) Risk of Bias: sampling bias by attrition and missing follow up information, and 2)Directness: Both studies were conducted in non-occupational exposures [↑](#footnote-ref-6)
7. Limitations/ loss of confidence due to: 1) Risk of Bias: Three studies^7,8,11^ are subject to sampling bias due to loss to follow-up, 2) Precision: Six studies^3-7,11^ have a small sample size or included a small number of participants with a source patient with a known undetectable HIV viral load. 3) Indirectness: Six studies^6-11^ are conducted in non-occupational populations (N = 1,998) [↑](#footnote-ref-7)
